# Amygdala neural ensemble mediates mouse social investigation behaviors

**DOI:** 10.1093/nsr/nwac179

**Published:** 2022-08-30

**Authors:** Ji-an Wei, Qing Han, Zhihua Luo, Linglin Liu, Jing Cui, Jiahui Tan, Billy K C Chow, Kwok-Fai So, Li Zhang

**Affiliations:** Key Laboratory of CNS Regeneration (Ministry of Education), Guangdong-Hong Kong-Macau Institute of CNS Regeneration, Jinan University, Guangzhou 510632, China; School of Biological Sciences, The University of Hong Kong, Hong Kong, China; Key Laboratory of CNS Regeneration (Ministry of Education), Guangdong-Hong Kong-Macau Institute of CNS Regeneration, Jinan University, Guangzhou 510632, China; Key Laboratory of CNS Regeneration (Ministry of Education), Guangdong-Hong Kong-Macau Institute of CNS Regeneration, Jinan University, Guangzhou 510632, China; Key Laboratory of CNS Regeneration (Ministry of Education), Guangdong-Hong Kong-Macau Institute of CNS Regeneration, Jinan University, Guangzhou 510632, China; Key Laboratory of CNS Regeneration (Ministry of Education), Guangdong-Hong Kong-Macau Institute of CNS Regeneration, Jinan University, Guangzhou 510632, China; Key Laboratory of CNS Regeneration (Ministry of Education), Guangdong-Hong Kong-Macau Institute of CNS Regeneration, Jinan University, Guangzhou 510632, China; School of Biological Sciences, The University of Hong Kong, Hong Kong, China; Key Laboratory of CNS Regeneration (Ministry of Education), Guangdong-Hong Kong-Macau Institute of CNS Regeneration, Jinan University, Guangzhou 510632, China; State Key Laboratory of Brain and Cognitive Science, Li Ka Shing Faculty of Medicine, The University of Hong Kong, Hong Kong, China; Center for Brain Science and Brain-Inspired Intelligence, Guangdong-Hong Kong-Macao Greater Bay Area, Guangzhou 510030, China; Bioland Laboratory (Guangzhou Regenerative Medicine and Health Guangdong Laboratory), Guangzhou 510006, China; Co-Innovation Center of Neuroregeneration, Nantong University, Nantong 220619, China; Neuroscience and Neurorehabilitation Institute, University of Health and Rehabilitation Sciences, Qingdao 266113, China; Institute of Clinical Research for Mental Health, Jinan University, Guangzhou 510632, China; Key Laboratory of CNS Regeneration (Ministry of Education), Guangdong-Hong Kong-Macau Institute of CNS Regeneration, Jinan University, Guangzhou 510632, China; Center for Brain Science and Brain-Inspired Intelligence, Guangdong-Hong Kong-Macao Greater Bay Area, Guangzhou 510030, China; Bioland Laboratory (Guangzhou Regenerative Medicine and Health Guangdong Laboratory), Guangzhou 510006, China; Neuroscience and Neurorehabilitation Institute, University of Health and Rehabilitation Sciences, Qingdao 266113, China; Institute of Clinical Research for Mental Health, Jinan University, Guangzhou 510632, China

**Keywords:** social interaction, basolateral amygdala, secretin

## Abstract

Innate social investigation behaviors are critical for animal survival and are regulated by both neural circuits and neuroendocrine factors. Our understanding of how neuropeptides regulate social interest, however, is incomplete at the current stage. In this study, we identified the expression of secretin (SCT) in a subpopulation of excitatory neurons in the basolateral amygdala. With distinct molecular and physiological features, BLA^SCT+^ cells projected to the medial prefrontal cortex and were necessary and sufficient for promoting social investigation behaviors, whilst other basolateral amygdala neurons were anxiogenic and antagonized social behaviors. Moreover, the exogenous application of secretin effectively promoted social interest in both healthy and autism spectrum disorder model mice. These results collectively demonstrate a previously unrecognized group of amygdala neurons for mediating social behaviors and suggest promising strategies for social deficits.

## INTRODUCTION

Both humans and rodents display social interest toward conspecific individuals [1–3]. Such innate social behaviors are mediated by both neural circuits [4–6] and neuroendocrine factors [7–9]. Various brain regions, including the prefrontal cortex [6,10–13] and subcortical nuclei such as the hippocampus [14,15], ventral tegmental area (VTA) [5,16] and hypothalamus [17,18], provide neural substrates for social behaviors. As a pivotal region integrating emotional information, the amygdala’s nuclei have also been implicated in social interactions [19,20]. For example, the activation of the medial amygdala (MeA) suppresses social interest [21,22], and the negative regulation of social engagement [23–25] has been reported in the basolateral amygdala (BLA). However, one cannot exclude the effect of negative emotional status on social inhibition due to the widely accepted function of the BLA in stress response and anxiety coding [26]. Further dissection of the molecular and cellular composition of the BLA during social engagement is therefore required.

A recently identified computational model proposed the coding of social exploration by an activity-defined neuronal ensemble of the mouse BLA [27]. These results, in conjunction with clinical evidence supporting the role of the BLA in human social experiences [28,29], raise one plausible model, as distinct neuronal subpopulations of the BLA may regulate unique behavioral modules including social investigation. Specific molecular markers are expected for neuronal subtyping to address this question. Besides neurotransmitters, neuropeptides are frequently proposed to define distinct neuronal subpopulations with specific neuronal circuits and behavioral modules, such as the cholecystokinin (CCK)+ cells of the BLA in mediating depressive-like behaviors [30]. Here we focused on secretin (SCT), a classical gut-peptide hormone that was initially identified in duodenal tissues for stimulating pancreatic secretion [31]. Recent advances also revealed the expression of SCT across multiple brain regions, including the cerebellum, hippocampus, hypothalamus and amygdala [32]. The receptor of SCT has also been found across the brain stem, limbic system and hypothalamic nuclei [33]. SCT thus potentially acts as one neuropeptide hormone executing both physiological and behavioral modulatory functions. Previous findings showed the role of SCT in the central regulation of food [34] or water [35] intake, as well as in modulating spatial memory [36]. It is worth noting that the activation of supraoptic neurons by SCT facilitates social recognition in rodents [37], and the potential value of SCT in ameliorating social deficits in autistic spectrum disorder (ASD) patients has been debated [38–40]. We thus investigated the molecular and circuitry mechanisms of SCT in mediating social behaviors.

In the current study, by generating an SCT-Cre knock-in mouse line, we identified a distinct subpopulation of excitatory BLA neurons that express SCT. Those BLA^SCT+^ cells display the same transcriptomic profiles as neuroendocrine cells, and specifically facilitate social investigation behaviors toward an unfamiliar, same-sex mouse while other BLA cells are anxiogenic and can antagonize such social interest. The distinction between these two BLA neuronal subtypes also resides in the cell-type-specific projecting patterns, as BLA^SCT+^ neurons preferentially activate the medial prefrontal cortex (mPFC) for facilitating social affiliations. Moreover, this amygdala-prefrontal circuit can be activated by SCT, which helps to attenuate social deficits in an ASD model. Our results collectively demonstrate an amygdala-prefrontal pathway to elicit social behaviors, and suggest a promising intervention strategy targeting social deficits.

## RESULTS

### SCT is expressed in the BLA and mediates innate social behaviors

**Figure 1.**
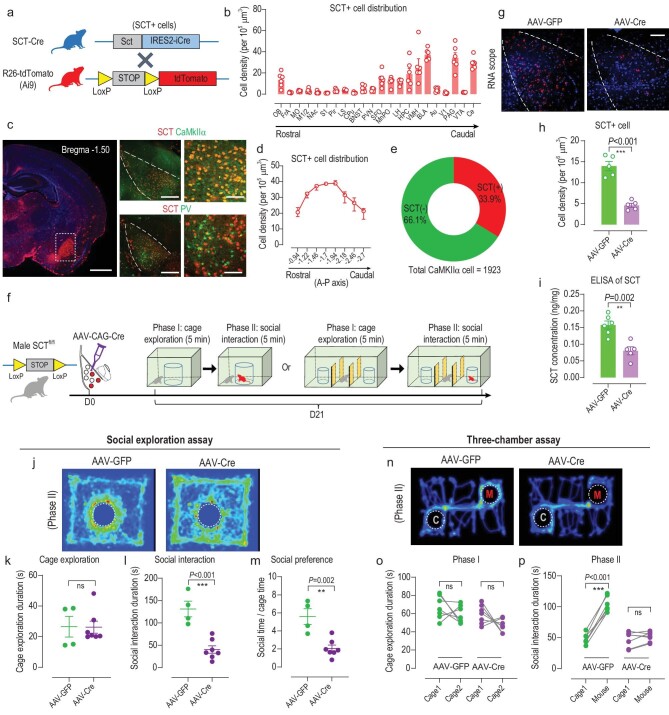
SCT expression and social-related functions. (a) Schematic diagram for generating the SCT-reporter mouse line. SCT-Cre mice were mated with R26-tdTomato (Ai9) mice to remove the STOP codon leading the fluorophore expression, thus labeling SCT-expressing cells only. (b) Spatial expressional profile of SCT as reflected by cell density across various brain regions (along the rostral-caudal axis). OB, olfactory bulb; FrA, frontal association cortex; MO, medial orbitofrontal cortex; M1/2, primary/secondary motor cortex; NAc, nucleus accumbens; S1, primary somatosensory cortex; Pir, piriform cortex; LS, lateral septum; CPu, caudate putamen; BNST, bed nucleus of the stria terminalis; PVN, paraventricular nucleus; SFO, subfornical organ; MnPO, median preoptic nucleus; LH, lateral hypothalamus; HPC, hippocampus; VMH, ventromedial hypothalamus; BLA, basolateral amygdala; Au, auditory cortex; V1, primary visual cortex; PAG, periaqueduct gray; VTA, ventral tegmental area; Ce, cerebellum. *n* = 6 animals (averaged from 4 slices per animal). (c) SCT expression in the BLA. Left panel, coronal section showing enrichment of SCT in the BLA. Scale bar, 500 μm. Right panels, co-labeling of SCT+ cells with CaMKIIα (top) or parvalbumin (bottom). Scale bar, 250 μm in pan-BLA view, and 100 μm in enlarged insets. (d) Density of SCT+ cells in the BLA along the rostral-caudal axis. *n* = 4 animals (averaged from 4 slices per animal). (e) Percentage of BLA^SCT+^ cells in total CaMKIIα+ cells in the BLA. (f) Experimental design of BLA-specific SCT gene knock-down assay. Male homozygous SCT-floxed (SCT^fl/fl^) mice received AAV-CAG-Cre injection into the bilateral BLA region, followed by (juvenile) social exploration assay or 3-chamber assay 3 weeks later. The social interaction test consisted of two phases. During the first 5-min phase, the mouse was placed into a novel field with an empty round cage in the center. For the second phase (5 min), one conspecific and novel mouse was placed into the central cage. Movement paths of the test mouse were recorded and analyzed for the time spent sniffing the central cage (mouse). The 3-chamber assay followed standard protocols (as described in Methods—Behavioral procedures), with 5 min in phase I and phase II. (g) RNA Scope images for the transcript of SCT in the BLA after SCT knock-down. Scale bar, 150 μm. (h) The quantification of RNA Scope showed decreased density of BLA^SCT+^ cells after gene knock-down. Two-sample unpaired *t*-test, *t*(8) = 7.745, *P* < 0.001. *n* = 6 mice per group. (i) ELISA for SCT in BLA tissues found lower peptide levels in the knock-down group. *t*(9) = 4.513, *P* = 0.002. *n* = 6 and 5 mice in AAV-GFP and AAV-Cre groups, respectively. (j) Heat maps showing relative place preference of mice during the (juvenile) social exploration assay. (k) No significant change in cage exploration time (during phase I). *t*(9) = 0.046, *P* = 0.964. (l) Decreased social interaction time after site-specific SCT gene knock-down. *t*(9) = 5.346, *P* < 0.001. (m) Lower social preference ratio (= social time/cage exploration time) in SCT knock-down group. *t*(9) = 4.422, *P* = 0.002. *n* = 4 and 7 mice in AAV-GFP and AAV-Cre groups, respectively, in (k–m). (n) Heat maps for mouse movement path during phase II of the 3-chamber assay. C, empty cage; M, novel mouse. (o) Both groups presented minor place preference when two empty cages were placed. Paired *t*-test, AAV-GFP group, *t*(6) = 1.088, *P* = 0.318; AAV-Cre group, *t*(6) = 1.840, *P* = 0.115. (p) During phase II when one novel mouse was placed, AAV-Cre mice (*t*(6) = 1.372, *P* = 0.219) lost social interest compared to AAV-GFP controls (*t*(6) = 15.22, *P* < 0.001). *n* = 6 mice per group in (o and p). ns, no significant difference; **P < 0.01; ***P < 0.001. All data are presented as mean ± sem.

To fully characterize the spatial expression pattern of SCT, we generated an SCT-IRES-Cre knock-in mouse line (Fig. S1a and b). By crossing SCT-Cre mice with a second reporter line (Ai9, R26-tdTomato), the whole-brain expressional profile of SCT was revealed (Fig. S1c). In general, SCT was minimally expressed in cortical regions, but with relatively high levels across various subcortical nuclei in the limbic system, midbrain and hindbrain (Fig. S1d and e). The specificity of SCT-Cre was further revealed by the localization of SCT transcript in tdTomato + cells (Fig. S1f). In particular, extensive distributions of SCT were found in the periaqueductal gray (PAG), ventral medial hypothalamus (VMH) and BLA, in addition to the cerebellum (Fig. 1a and b). These SCT-expressing neurons in the BLA (BLA^SCT+^) lay across the whole amygdala nuclei and occupy 33.9% of all CaMKIIα+ neurons but not parvalbumin (PV) interneurons (Fig. 1c–e). These data collectively suggest the enrichment of SCT in a subpopulation of excitatory neurons in the BLA.

Having observed the existence of SCT in the BLA, we next investigated its behavioral implications. By stereotaxic injection of an adeno-associated virus (AAV) carrying Cre recombinase into male homozygous SCT-floxed mice (SCT^fl/fl^), we performed a battery of behavioral screenings and found no significant change of anxiety behaviors or fear conditioning in those conditional SCT knock-out mice (Fig. S2a–e). These results suggest that SCT seems to be irrelevant to those well-known functions of the BLA. When we adopted a social exploration task in which the test mouse was allowed to visit an unfamiliar male mouse (4 weeks old) in the center of a novel field, however, the downregulation of SCT in the BLA (Fig. 1f–i) suppressed mouse social interest remarkably (Fig. 1j). Since the exploration behavior toward an empty cage was not affected by SCT knock-down (Fig. 1k), the decreased social interaction time and social preference ratio (= social time/cage exploration time) faithfully reflected impaired social investigation interest rather than the carry-on effect by potential anxiolysis (Fig. 1l and m). To further validate the social modulation of SCT, we recruited the classical 3-chamber assay and found that during phase II (with one novel mouse and one empty cage), SCT knock-down deprived normal social preference (Fig. 1n–p). Moreover, in female mice with SCT knock-down, social investigation toward an unfamiliar female conspecific was also repressed in both an open field and 3-chamber apparatus (Fig. S2f–k), illustrating the robust role of SCT in modulating social exploration behaviors across genders. Therefore, SCT in the BLA is necessary for innate social approaching behaviors.

### BLA^SCT+^ neurons positively mediate social interaction

The behavioral phenotyping of SCT knock-down assays seems to be incompatible with current knowledge, which claims the suppressive effect of social interaction by BLA projecting neurons [23–25]. But the existence of SCT in only one third of excitatory neurons of the BLA (Fig. 1e) leaves one possible explanation: that SCT represents a distinct group of BLA cells that specifically facilitates social interest. To test this, we firstly performed a cell-ablation assay to selectively erase BLA^SCT+^ neurons by locally infecting an AAV vector encoding Caspase3 under the double-floxed inverted open reading frame (DIO) scheme (Fig. S3a–c). The decreased social exploration time (Fig. S3e and f) suggests that these cells were probably involved in social investigation behaviors, although the decrement of cage exploration time (Fig. S3d) also indicated the multifaceted function of BLA^SCT+^ neurons.

**Figure 2.**
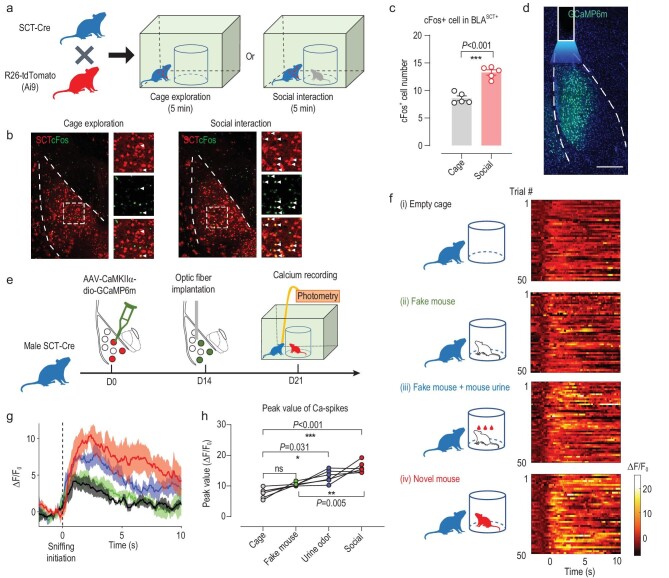
BLA^SCT+^ cell activity correlates with social behaviors. (a) Schematic diagram for cFos quantification. SCT-Cre; Ai9 reporter mice were allowed a 5-min session of empty-cage exploration or social interaction. After 60 min, the whole brain was dissected for immunofluorescent staining. (b) Representative images for BLA^SCT+^ cells and cFos activity. More co-labeling can be found in the social interaction group (white arrowheads). Scale bars, 150 μm in the pan-BLA view, and 50 μm in the enlarged insets. (c) Quantification analysis showed higher cFos activity in BLA^SCT+^ neurons after social interaction. Two-sample unpaired *t*-test, *t*(8) = 7.202, *P* < 0.001. *n* = 5 mice in each group (averaged from 4 slices per animal). (d) Experimental design of *in vivo* calcium recording of BLA^SCT+^ neurons. SCT-Cre mice were unilaterally infused with AAV-dio-GCaMP6m, followed by fiber implants 2 weeks later. On day 21 post-injection, the fiber photometry apparatus was adopted to record populational activity in behavior. (e) Sample slices showing the GCaMP6m infection and fiber implantation sites. Scale bar, 250 μm. (f) Heat maps of calcium transients under different grades of social cues. A total of 50 trials were presented for each group. All recording traces were realigned with respect to the initiation of sniffing behaviors (time 0). (g) Normalized calcium transients (in Δ*F*/*F*_0_) from different treatment groups. The solid curve represents averaged values, and the shaded area reflects population sem. (h) Peak values of calcium transients were gradually increased when the test mouse faced stronger social cues. One-way analysis of variance (ANOVA) with repeated measures, *F* (1.332, 6.660) = 26.51, *P* = 0.001; Tukey's post-hoc comparisons: Cage vs. Fake mouse, *P* = 0.063; Cage vs. Urine odor, *P* = 0.031; Cage vs. Social, *P* < 0.001; Fake mouse vs. Social, *P* = 0.005. *n* = 6 mice, with 10 trials under each stimulus. *P < 0.05; **P < 0.01; ***P < 0.001. All data are presented as mean ± sem.

To better correlate the cellular activity of BLA^SCT+^ neurons with social behaviors, we quantified the immediate early gene (IEG) cFos expression after a brief social exploration session using SCT-Cre; Ai9 reporter lines (Fig. 2a). Compared with mice encountering an empty cage, sociable mice presented more activated BLA^SCT+^ cells (Fig. 2b and c). An *in vivo* recording assay was subsequently performed by implanting an optic fiber into the BLA of a SCT-Cre mouse whose SCT+ cells were infected with genetically encoded calcium indicator GCaMP6m (Fig. 2d and e). Using the fiber photometry apparatus, the population activity of BLA^SCT+^ cells was recorded when the animal faced different grades of social cues (empty cage, fake mouse, fake mouse with urine from a novel mouse, and novel animal; see Fig. 2f). In general, those cells showed significantly elevated calcium peaks when facing the urine odor of an unfamiliar mouse but not the fake mouse, and the strongest response occurred during the actual social interaction scenario (Fig. 2g and h). Those results provide *in vivo* evidence supporting the coding of social cues by BLA^SCT+^ cells.

**Figure 3.**
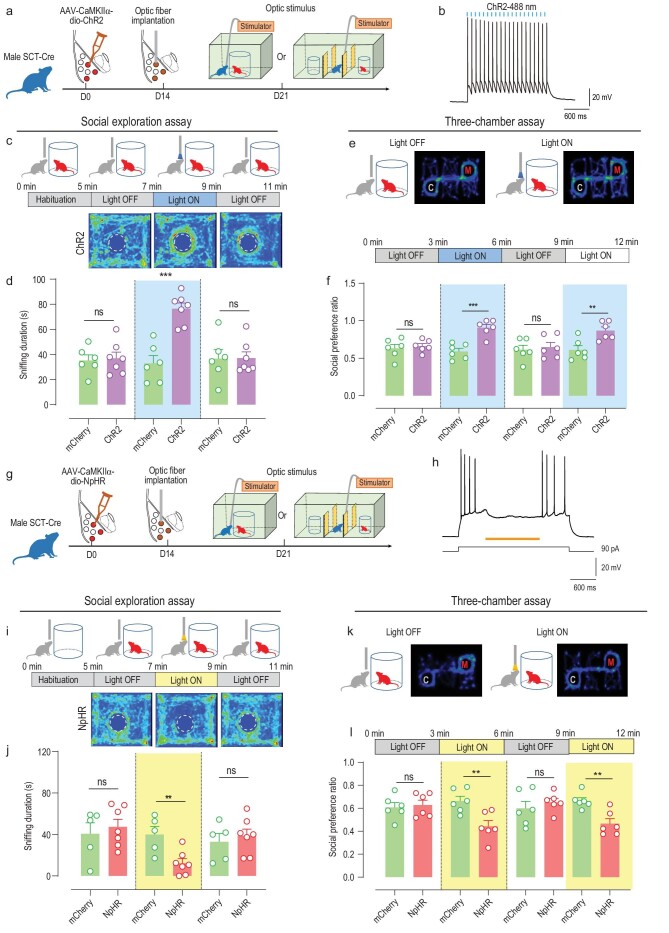
BLA^SCT+^ neurons facilitate social interaction. (a) Schematic diagram of optogenetics activation of BLA^SCT+^ neurons. Excitatory channelrhodopsin-2 (ChR2) was introduced into BLA^SCT+^ cells and stimulating optic fibers were implanted into the bilateral BLA. One week after fiber implantation, the mouse was adopted in the social behavior paradigm under light stimulation. (b) *Ex vivo* electrophysiological recordings confirmed the effectiveness of ChR2 in modulating neuronal activities. (c) Experimental design (top) and heat map (bottom) of social exploration assays under light activation. The test session consisted of three test sessions (2 min duration each) in which a stimulation session (Light ON) was flanked by two interval sessions (Light OFF), following a 5-min habituation phase. (d) Light activation of BLA^SCT+^ cells enhanced social behaviors, which were rapidly diminished when light stimulus was lifted. Unpaired *t*-test, phase 1 (Light OFF), *t*(11) = 0.2645, *P* = 0.796; phase 2 (Light ON), *t*(11) = 5.766, *P* < 0.001; phase 3 (Light OFF), *t*(11) = 0.0556, *P* = 0.957. *n* = 6 and 7 mice in mCherry and ChR2 groups, respectively. (e) Heat maps of mice during the 3-chamber assay (with one novel mouse plus one cage) under light stimulus. (f) Mice presented enhanced social interest toward the novel mouse under light stimulus and decreased social preference ratio with the removal of light stimulation. Phase 1 (Light OFF), *t*(10) = 0.4804, *P* = 0.641; phase 2 (Light ON), *t*(10) = 5.229, *P* < 0.001; phase 3 (Light OFF), *t*(10) = 0.4210, *P* = 0.683; phase 4 (Light ON), *t*(10) = 3.464, *P* = 0.006. *n* = 6 mice per group. (g) Schematic diagram of the optogenetics inhibition of BLA^SCT+^ neurons using halorhodopsin (NpHR). The experimental design was the same as in (a). (h) *Ex vivo* electrophysiological recordings confirmed the effectiveness of NpHR in modulating neuronal activities. (i) Experimental flow of light inhibition assay (top) and heat maps (bottom) during the social exploration assay. The outflow of the assay was the same as in (c). (j) Light inhibition of BLA^SCT+^ cells suppressed social interactions. Phase 1 (Light OFF), *t*(10) = 0.5469, *P* = 0.596; phase 2 (Light ON), *t*(10) = 3.348, *P* = 0.007; phase 3 (Light OFF), *t*(10) = 0.5509, *P* = 0.594. *n* = 5 and 7 mice in mCherry and ChR2 groups, respectively. (k) Heat maps of mice during the 3-chamber assay (with one novel mouse plus one cage) under light stimulus. (l) Mice presented depressed social interest toward the novel mouse under light stimulus and recovery of normal social preference ratio when the light was lifted. Phase 1 (Light OFF), *t*(10) = 0.3405, *P* = 0.741; phase 2 (Light ON), *t*(10) = 3.418, *P* = 0.007; phase 3 (Light OFF), *t*(10) = 0.7657, *P* = 0.462; phase 4 (Light ON), *t*(10) = 3.905, *P* = 0.003. *n* = 6 mice per group. ns, no significant difference; **P < 0.01; ***P < 0.001. All data are presented as mean ± sem.

The role of BLA^SCT+^ neurons in social interaction was further tested by a cell-type-specific manipulation strategy in which channelrhodopsin-2 (ChR2) was expressed in those cells (Fig. 3a and b). Under the same social exploration test, cell activation strongly potentiated social sniffing behaviors (Fig. 3c and d, Video S1). Similar phenotypes were observed in the 3-chamber assay, in which light activation potentiated mouse social preference, and the removal of optogenetic stimulus abolished such increment (Fig. 3e and f). On the other hand, optogenetic inhibition of BLA^SCT+^ neurons using halorhodopsin (NpHR; Fig. 3g and h) suppressed social interest, as the light stimulus remarkably inhibited the social interest of the test mouse (Fig. 3i and j). Three-chamber assays also displayed similar behavioral modulations, as inhibition of BLA^SCT+^ cells had diminished social interest, which was rapidly recovered after the removal of light stimulus (Fig. 3k and l). To rule out the possible effects of place preference or social-related rewards, we repeated the optogenetic excitation assay but with the removal of the novel mouse. The absence of social cues did not facilitate sniffing toward the empty cage, given the existence of prior social experience (Fig. S4a). Moreover, the activation of BLA^SCT+^ neurons did not cause a preference toward the empty cage (Fig. S4b). These real-time neural manipulation assays agreed with prior *in vivo* recording results (Fig. 2), and support both the necessary and sufficient role of BLA^SCT+^ cells in modulating social investigation behaviors. As further evidence, we also performed chemogenetic assays by introducing designer receptors exclusively activated by designer drugs (DREADDs) hM3Dq or hM4Di into BLA^SCT+^ neurons (Fig. S5a and b). The infusion of receptor ligand clozapine-N-oxide (CNO) rapidly potentiated social investigation behaviors in hM3Dq-infected animals (Fig. S5c–f) or suppressed social interest toward the novel animal when hM4Di was expressed in BLA^SCT+^ neurons (Fig. S5g–j). In sum, the BLA^SCT+^ neural ensemble is activated by social cues and facilitates innate social investigation behaviors.

### SCT defines two distinct BLA neuronal subpopulations

**Figure 4.**
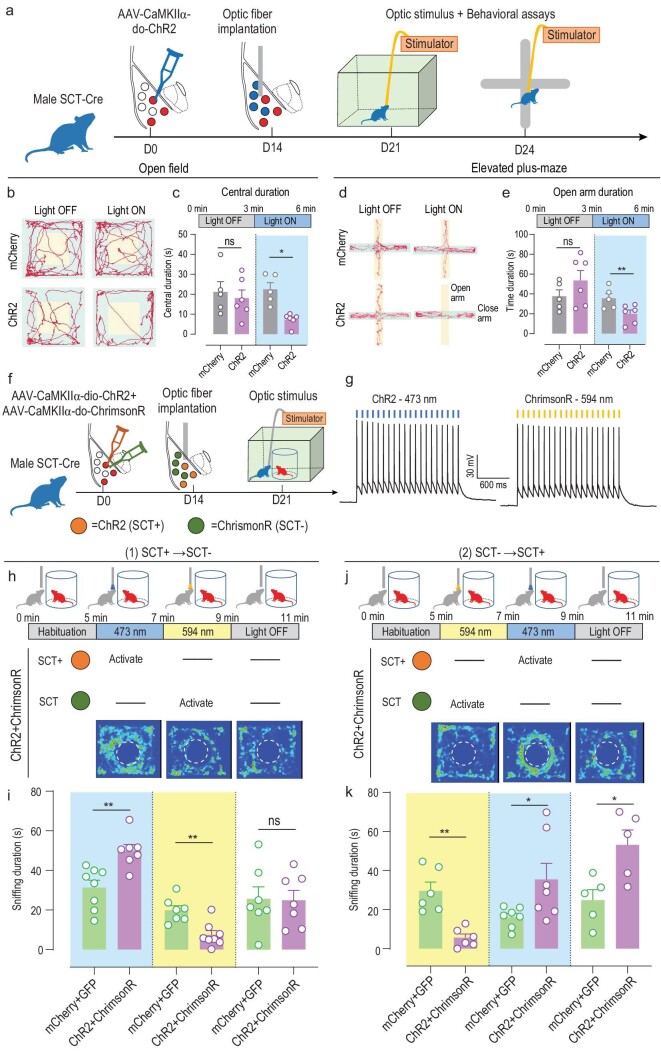
BLA^SCT+^ and BLA^SCT−^ neurons differentially regulate social interactions. (a) Schematic illustrations for labeling and manipulating BLA^SCT−^ cells. Similar to Fig. 3a, mice received infection of AAV-do-ChR2 and optic fiber implantation, and behavioral tests were performed 3 weeks later. (b) Movement paths in the open field arena, in which ChR2-infected mice preferred to stay in the peripheral region during the light stimulation. (c) Optogenetic activation of BLA^SCT−^ cells decreased central duration. One-way ANOVA, *F*(3, 18) = 3.725, *P* = 0.030. Tukey's post-hoc comparison between mCherry and ChR2: phase 1 (Light OFF), *P* = 0.929; phase 2 (Light ON), *P* = 0.039. (d) Movement paths in the elevated plus maze, in which ChR2-infected mice avoided the entry to the open arms during the light stimulation. (e) The activation of BLA^SCT−^ cells resulted in shorter open-arm duration. One-way ANOVA, *F*(3, 18) = 4.340, *P* = 0.018. Tukey's post-hoc comparison between mCherry and ChR2: phase 1 (Light OFF), *P* = 0.394; phase 2 (Light ON), *P* = 0.001. *n* = 5 and 6 mice in mCherry and ChR2, respectively, in (b–e). (f) Experimental flowchart of dual-colored light stimulation of BLA^SCT+^ and BLA^SCT−^ cells. ChR2 and ChrimsonR were infected into SCT-Cre mice under dio- and do- schemes, respectively. Fiber implantation and behavioral assays were the same as (a). (g) Two different stimulation wavelengths (473 nm and 594 nm) specifically activated ChR2+ and ChrimsonR+ cells, respectively. (h) Behavioral assay protocols (top) and heat map (bottom) of light activation assays. The test mouse was sequentially treated with 473 nm light, 594 nm light and a Light OFF session (2 min duration each). (i) Light activation of BLA^SCT+^ cells enhanced social behaviors whilst activation of BLA^SCT−^ cells inhibited social behaviors. One-way ANOVA, *F*(5, 38) = 12.92, *P* < 0.001. Tukey post-hoc comparison, mCherry+ GFP vs. ChR2+ ChrimsonR: phase 1 (473 nm excitation), *P* = 0.002; phase 2 (594 nm excitation), *P* = 0.004; phase 3 (Light OFF), *P* > 0.999. *n* = 8 and 7 mice in mCherry+ GFP and ChR2+ ChrimsonR group, respectively. (j) Top, behavioral assays for the reverse-ordered light excitation, in which the test mouse sequentially received 594 nm stimulation, 473 nm stimulation and no light. Bottom, representative heat maps of mouse tracks. (k) Light activation of BLA^SCT−^ neurons suppressed social interactions, followed by BLA^SCT+^ cell activation-induced social affiliations, which again presented a latent effect in the Light OFF phase. One-way ANOVA, *F*(5, 30) = 8.237, *P* < 0.001. Tukey post-hoc comparison, mCherry+ GFP vs. ChR2+ ChrimsonR: phase 1 (594 nm excitation), *P* = 0.002; phase 2 (473 nm excitation), *P* = 0.038; phase 3 (Light OFF), *P* = 0.032. *n* = 6 mice in each group. ns, no significant difference; *P < 0.05; **P < 0.01; ***P < 0.001. All data are presented as mean ± sem.

The demonstration of BLA^SCT+^ cells in mediating social interactions cannot fully support the specificity of such a neural ensemble in behavioral modulation, as other excitatory neurons in the BLA (BLA^SCT−^) may also participate in this process. To address this question, we designed a second set of AAV vectors carrying CaMKIIα-ChR2 under a double-floxed orientation (DO) frame, whose expression can be turned off only by the presence of Cre recombinase [41–43] (Fig. 4a). After expressing ChR2 in BLA^SCT−^ cells, a set of behavioral tests found that the activation of BLA^SCT−^ cells induced anxiety-like behaviors (Fig. 4b–e), agreeing with our current knowledge of the BLA in the anxiogenic process. We further propose that BLA^SCT−^ cells may play different roles in social interaction in contrast with BLA^SCT+^ cells. To further validate the antagonistic effects of two BLA subpopulations, we co-infected ChR2 and a second excitatory optogenetic receptor, ChrimsonR, with different excitation spectra [44], into the BLA of SCT-Cre mice, under DIO and DO flanked sequences, respectively (Fig. 4f). Such experimental design successfully implanted a dual-switch system in which BLA^SCT+^ neurons were activated by 473 nm excitation light, and BLA^SCT−^ cells were excited under 594 nm wavelength stimulation (Fig. 4g). During the social approaching paradigm, the sequential activation of two neuronal subtypes produced opposite effects on social sniffing duration (Fig. 4h and i, Video S2). Specifically, ChR2 stimulation on BLA^SCT+^ facilitated social interest, whilst ChrimsonR excitation on BLA^SCT−^ inhibited social behaviors. Such phenotypes are stable despite the change of stimulating sequence (Fig. 4j and k), suggesting the cell specificity of these two BLA subpopulations for antagonistic modulation of social interest.

The distinct behavioral modules further implied possibly unique molecular features between BLA^SCT+^ and BLA^SCT−^ cells. To address this issue, we firstly studied the possible overlap between SCT and known molecular markers of BLA cells. The BLA^SCT+^ cells were mostly co-localized with Ppp1r1b (Fig. S6), which represents a group of BLA cells encoding positive valence [45]. However, as the BLA^SCT+^ cells only represent a fraction of Ppp1r1b + cells, we further characterized those neurons by infecting the SCT-Cre; Ai9 reporter line with AAV-CaMKIIα-DO-GFP, resulting in dual fluorescent labels on BLA^SCT+^ and BLA^SCT−^ cells (Fig. S7a). A preliminary examination found minimal overlapping between GFP+ and tdTomato+ labeled cells, as BLA^SCT+^ cells mainly occupy the medial region whilst BLA^SCT−^ cells are predominantly located in the lateral part (Fig. S7b). The divergent spatial distribution further implies unique molecular, physiological and anatomic features of these two cell types. We thus performed a single-cell-based transcriptomic study after extracting cellular contents via a glass pipette (Fig. S7a). Based on data from a total of 27 neurons, the principal component analysis (PCA) showed that BLA^SCT−^ and BLA^SCT+^ cells belonged to two distinct groups with unique transcriptomic profiles (Fig. S7c). Of note, a total of 2440 genes were differentially expressed (Figs S7d and S8). Gene enrichment analysis indicated that these genes were involved in neuropeptide ligand-receptor binding and intracellular pathways such as cAMP (Fig. S7e and f). Those molecular features further implied distinctions of cellular properties. *Ex vivo* electrophysiological recording showed that BLA^SCT+^ cells exhibited higher excitability, lower input resistance and lower rheobase compared to their counterparts without SCT expression (Fig. S7g–j), in conjunction with altered membrane properties (Fig. S9a and b) but with their action potential (AP) kinetics left unchanged (Fig. S9c–h). Those comparative studies demonstrated that BLA^SCT+^ represented a unique group of excitatory neurons that specifically mediates social investigation behaviors.

### BLA^SCT^ neurons drive an amygdala-prefrontal circuit to regulate social behaviors

**Figure 5.**
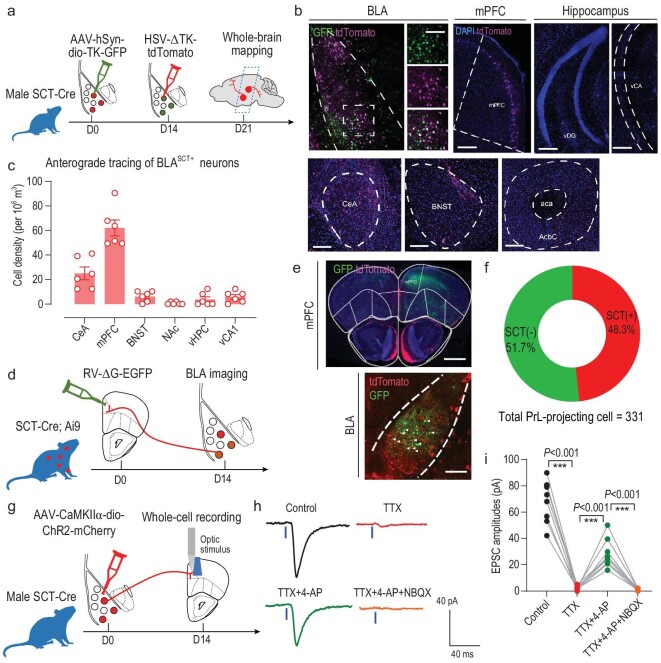
BLA^SCT+^ cells form monosynaptic excitatory projection to the mPFC. (a) Schematic illustrations of anterograde tracing of BLA^SCT+^ neurons. SCT-Cre mice were sequentially infected with a helper virus (AAV-dio-TK-GFP) and a mutant herpes simplex virus (HSV-ΔTK-tdTomato). The projecting targets of BLA^SCT+^ neurons were visualized in (b), and were quantified by (c). *n* = 5 mice per group (averaged from 4 slices per animal). (d) Retrograde tracing of mPFC inputs. The SCT-Cre; Ai9 reporter line was infected with a mutant form of rabies virus (RV-ΔG-EGFP), and the input cell soma was examined in the BLA. (e) RV injection sites in the mPFC (left) and input cells in the BLA (right). A significant number of mPFC-projecting cells in the BLA were co-labeled with SCT (white arrowheads). Scale bar, 500 μm in left, and 150 μm in right. (f) Percentage of SCT+ cells in mPFC-projecting neurons. A total of 351 prelimbic (PrL)-projecting cells were enumerated from three mice. (g) Experimental schedules of *ex vivo* optogenetics manipulation of the BLA^SCT+^→mPFC terminus. BLA^SCT+^ neurons were infected with ChR2, followed by acute brain slice preparation and whole-cell recording, coupled with light stimulus 2 weeks later. (h) Sample traces of mPFC neurons showed rapid depolarization upon light activation at the BLA^SCT+^ axonal terminus, and the suppressed postsynaptic response by tetrodotoxin. Moreover, the light-induced action potentials can be recovered by adding 4-aminopyridine (4-AP) into the perfusion solution and were totally blocked by 2,3-dihydroxy-6-nitro-7-sulfamoyl-benzoquinoxaline-2,3-dione (NBQX). (i) Sampled quantification of (h). One-way ANOVA, *F*(3,32) = 102.8, *P* < 0.001. Tukey's post-hoc comparisons: control vs. TTX, TTX vs. TTX+4-AP, TTX+4-AP vs. TTX+4-AP+NBQX; *P* < 0.001 in all. A total of nine neurons from three mice were recorded and analyzed. ***P < 0.001. All data are presented as mean ± sem.

In addition to the molecular and electrophysiological features, features in neural circuitry architectures may further help to address the differential behavioral modules between BLA^SCT+^ and BLA^SCT−^ cells. Using the AAV-CaMKIIα-DO-GFP vector-mediated infection on SCT-Cre mice (Fig. S10a), we found that BLA^SCT−^ neurons prominently extend their axonal terminus into the ventral CA1, NAc and bed nucleus of the stria terminalis (BNST) in addition to the central amygdala (CeA) (Fig. S10b and c), which agrees with previous findings [30,46–48]. However, when we examined the downstream target of BLA^SCT+^ neurons using an anterograde trans-synaptic herpes simplex virus (HSV) in conjunction with the Cre-mediated helper virus (Fig. 5a), those cells preferentially innerved the mPFC in addition to the proximal CeA, instead of subcortical projections (Fig. 5b and c). To validate the existence of this BLA→mPFC pathway, a modified rabies virus (RV) was infused into the prelimbic (PrL) of SCT-Cre; Ai9 reporter mice (Fig. 5d) to trace the input of those cells. In the BLA region, ∼48.3% of PrL-projecting BLA cells were co-labeled with SCT (Fig. 5e and f), highlighting the input of SCT+ cells in this circuit. The nature of this synaptic connection was further examined by patch-clamp recording of PrL neurons under *ex vivo* optogenetics stimulation, with prior ChR2 infection into BLA^SCT+^ neurons (Fig. 5g). The abolishment of excitatory postsynaptic current (EPSC) by tetrodotoxin (TTX) infusion, plus the rapid recovery by 4-aminopyridine (4-AP) all suggested the mono-synaptic excitatory transmission between BLA^SCT+^ and PrL cells (Fig. 5h and i).

**Figure 6.**
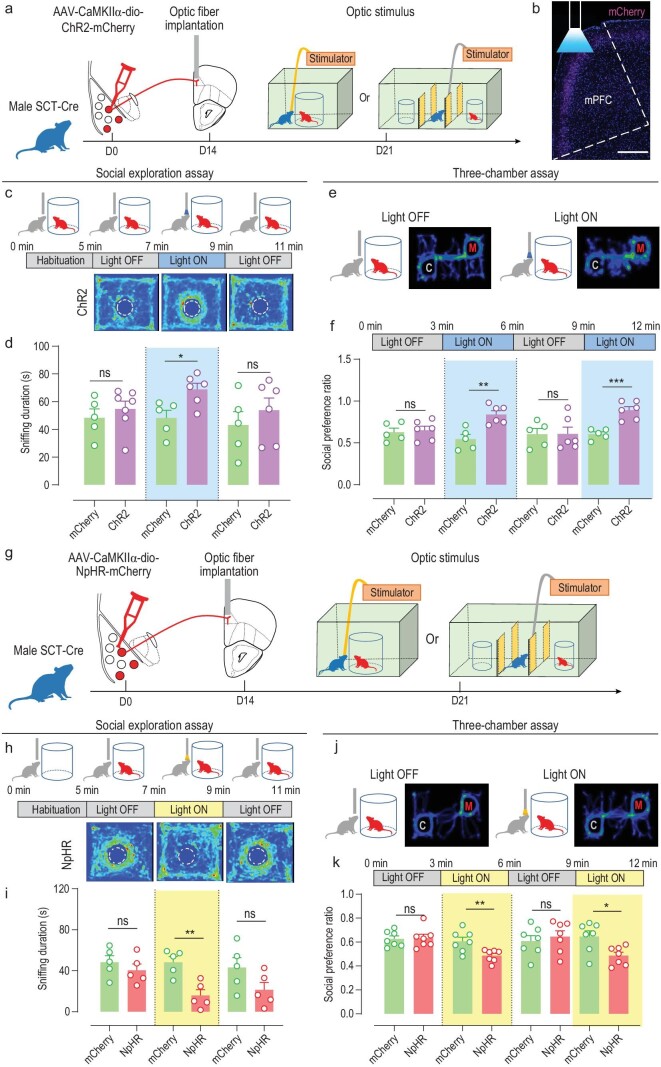
The BLA^SCT+^→mPFC pathway mediates social interaction behaviors. (a) Experimental schedules of optogenetics activation of the BLA^SCT+^→mPFC terminus. BLA^SCT+^ neurons were infected with ChR2, followed by fiber implantation into the PrL region 2 weeks later, and behavioral assays at D21. (b) The implantation site of the optic fiber for mPFC terminal stimulus or inhibition. Scale bar, 500 μm. (c) Behavioral phenotyping protocols (top, as in Fig. 3c) and heat maps for movement paths (bottom) under light stimulus during the social exploration assay. (d) Light activation of the BLA^SCT+^→mPFC terminus enhanced social sniffing behaviors but without latent effects. Two-sample unpaired *t*-test between mCherry and ChR2 groups: phase 1 (Light OFF), *t*(10) = 0.749, *P* = 0.471; phase 2 (Light ON), *t*(10) = 2.940, *P* = 0.017; phase 3 (Light OFF), *t*(10) = 0.810, *P* = 0.439. *n* = 6 mice in each group. (e) Heat maps of mice during the 3-chamber assay (with one novel mouse plus one cage) under light stimulus. (f) Mice presented enhanced social interest toward the novel mouse under light stimulus and decreased social preference ratio with the removal of light stimulation. Phase 1 (Light OFF), *t*(9) = 0.3483, *P* = 0.736; phase 2 (Light ON), *t*(9) = 4.156, *P* = 0.003; phase 3 (Light OFF), *t*(9) = 0.0321, *P* = 0.975; phase 4 (Light ON), *t*(9) = 5.254, *P* < 0.001. *n* = 5 and 6 mice in the mCherry and ChR2 groups, respectively. (g) Experimental schedules of optogenetics inhibition of the BLA^SCT+^→mPFC terminus. BLA^SCT+^ neurons were infected with NpHR and the rest of the design was the same as in (a). (h) Behavioral phenotyping protocols (top) and heat maps for movement paths (bottom) for the light inhibition assay. (i) Light inhibition of the BLA^SCT+^→mPFC terminus suppressed social behaviors. Phase 1 (Light OFF), *t*(8) = 0.903, *P* = 0.393; phase 2 (Light ON), *t*(8) = 4.126, *P* = 0.003; phase 3 (Light OFF), *t*(8) = 1.806, *P* = 0.109. *n* = 5 mice in each group. (j) Heat maps of mice during the 3-chamber assay (with one novel mouse plus one cage) under optogenetic inhibition. (k) Mice presented decreased social interest toward the novel mouse under light stimulus and the recovery of normal social preference after the removal of light stimulation. Phase 1 (Light OFF), *t*(12) = 0.1841, *P* = 0.857; phase 2 (Light ON), *t*(12) = 3.081, *P* = 0.010; phase 3 (Light OFF), *t*(12) = 0.5650, *P* = 0.583; phase 4 (Light ON), *t*(12) = 2.896, *P* = 0.013. *n* = 7 mice per group. ns, no significant difference; *P < 0.05; **P < 0.01; ***P < 0.001. All data are presented as mean ± sem.

Since the mPFC has been recognized as playing a role in social behaviors [11–13], we next tested if the BLA^SCT+^→mPFC pathway is relevant to the social interaction modulation. After infecting BLA^SCT+^ cells with Cre-dependent ChR2, we implanted an optic fiber into the mPFC region to stimulate the axonal terminus originating from the BLA (Fig. 6a). Using identical experimental protocols as those in somatic manipulation (Fig. 3), the activation of the BLA^SCT+^→mPFC terminus elongated social sniffing durations in the social exploration assay (Fig. 6c and d) or in the 3-chamber assay (Fig. 6e and f). Light-mediated inhibition of these fibers (Fig. 6g) also mimicked the effect of soma manipulation, suggesting the suppression of social interest (Fig. 6h–k). Combining all those data, it is likely that BLA^SCT+^ specifically facilitates innate social investigation behaviors primarily via their projections to the mPFC.

### Exogenous SCT facilitates social behaviors in naïve and ASD mice

**Figure 7.**
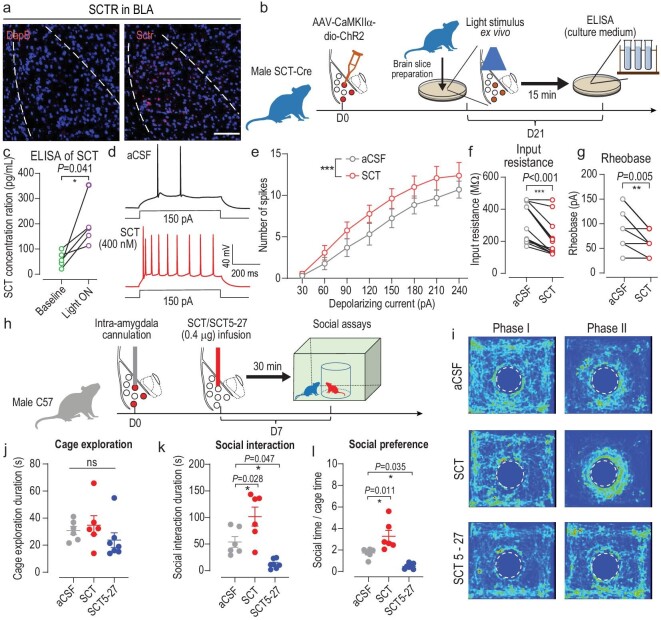
SCT activates BLA neurons and facilitates mouse innate social interactions. (a) RNAScope for secretin receptor (Sctr) expression in the BLA. Scale bar, 150 μm. (b) Schematic diagrams of experimental flow for SCT quantification. SCT-Cre mice were infected with AAV-dio-ChR2. Three weeks later, *ex vivo* optogenetic stimulation was performed on acute BLA slices. Culture medium was sampled before and at 15 min after light stimulation for ELISA. (c) SCT release in BLA tissues was remarkably increased after ChR2 activation, indicating that endogenous SCT release is activity-dependent. Paired *t*-test, *t*(5) = 2.986, *P* = 0.041. *n* = 5 mice per group. (d) Sample traces of electrophysiological recording on BLA neurons, with 400 nM SCT added in the perfusion buffer. (e) Higher number of spikes under the same injection current when BLA cells received SCT treatment, suggesting potentiated excitability. Two-way ANOVA with respect to group factor, *F*(1, 192) = 11.74, *P* < 0.001. (f) Input resistance was decreased when BLA neurons were treated with SCT. Two-sample paired *t*-test, *t*(12) = 5.354, *P* < 0.001. (g) Rheobase values were decreased after SCT perfusion. *t*(12) = 3.488, *P* = 0.005. *n* = 13 neurons from 4 mice in (e–g). (h) Experimental protocols for behavioral modulation assay of SCT. Male wild-type mice were bilaterally implanted with paired cannulas, through which 0.4 μg SCT or SCT 5–27 was administrated, followed by a social interaction assay after 30 min. (i) Sample heat maps reflecting the spatial preference of animals. (j) No change of cage exploration time under SCT or SCT 5–27 treatment. One-way ANOVA, *F*(2, 16) = 1.113, *P* = 0.353. (k) SCT increased social sniffing duration of wild-type mice whilst SCTR blockade by SCT 5–27 suppressed social behaviors. *F*(2, 16) = 6.383, *P* = 0.009. Tukey's post-hoc test: SCT vs. aCSF, *P* = 0.028; SCT 5-27 vs. aCSF, *P* = 0.047. (l) SCT modulates mouse social preference. *F*(2, 16) = 19.57, *P* < 0.001. Tukey's post-hoc test: SCT vs. aCSF, *P* = 0.011; SCT 5-27 vs. aCSF, *P* = 0.035. *n* = 6, 6 and 7 animals in aCSF, SCT and SCT 5–27 groups, respectively, in (j–l). ns, no significant difference; *P < 0.05; ***P < 0.001. All data are presented as mean ± sem.

Since BLA^SCT+^ population potentiates social interest, further questions are raised as to whether SCT peptide plays a role in social modulations. To elaborate the molecular mechanism of social behaviors, we firstly identified the prominent expression of SCT receptor (SCTR) transcript in the BLA region (Fig. 7a), providing the molecular substrate for locally SCT-mediating effects. In a second study using acute BLA slices from SCT-Cre mice infected with AAV-dio-ChR2, a brief light-mediated excitation of BLA^SCT+^ cells remarkably increased SCT release into a culture medium (Fig. 7b and c). These results indicated possible autocrine and/or paracrine mechanisms in which SCT was released from activated BLA^SCT+^ neurons to potentiate social behaviors. As direct evidence of cellular functions, SCT perfusion induced higher spiking numbers plus lower input resistance or rheobase values, in addition to altered kinetics of AP under *ex vivo* patch-clamp recording (Fig. 7d–g, Fig. S11), suggesting the direct neuromodulatory role of SCT. Taken together, SCT acts as the central neuromodulator to potentiate BLA cells for mediating social investigation behaviors.

To further establish the role of SCT in social behaviors, we introduced a recombinant SCT peptide or an SCTR antagonist (SCT 5–27) into the bilateral BLA of C57 wild type male mice via a pre-implanted cannula (Fig. 7h). Social approaching assay found that SCT administration remarkably increased social exploration time and social preference, whilst receptor blockade by SCT 5–27 remarkably suppressed social interest but left the cage exploration time unchanged (Fig. 7i–l). To test whether the facilitated sniffing behavior was dependent on social cues, we repeated this assay using an aggressive CD1 mouse in the central cage. Behavioral phenotyping showed that, with prior exposure to CD1, SCT infusion did not enhance the social duration (Fig. S12), suggesting that SCT only enhanced ‘amicable’ social investigation but did not affect the avoidance of formidable social cues. Moreover, we employed a chronic SCT administration scenario in which SCT expression was persistently driven by an AAV vector infected into the BLA (Fig. S13a and b). Similar to those phenotypes in SCT drug infusion, AAV-SCT infection remarkably increased social preference (Fig. S13c–e). On the other hand, the suppression of SCTR function using short hairpin RNA (shRNA) targeting SCTR also resulted in decreased social interest (Fig. S13f–i). Those data clearly suggested the indispensable role of the SCT-SCTR axis in innate social behaviors.

**Figure 8.**
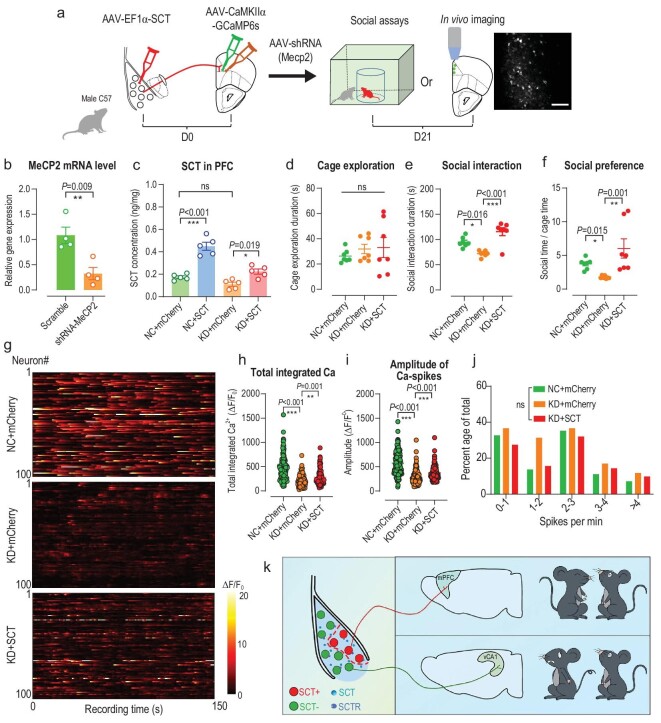
SCT relieves ASD-related social deficits via activation of the BLA-mPFC pathway. (a) Experimental procedures for behavioral modulation and *in vivo* recording assays. The mPFC of male wild-type mice was co-infected with AAV-shRNA (MeCP2) and AAV-GCaMP6s, and the BLA with AAV-SCT. Three weeks later, mice were adopted in a social interaction assay, or *in vivo* calcium recording. Scale bar in the field of view under 2-photon microscopy, 100 μm. (b) Relative expression level of MeCP2 in the mPFC suggested effectiveness of gene knock-down. Two-sample unpaired *t*-test, *t*(6) = 3.776, *P* = 0.009. *n* = 4 mice in each group. (c) Elevated SCT concentrations in PFC under viral infection. One-way ANOVA, *F*(3, 16) = 37.95, *P* < 0.001. *n* = 5 mice in each group. (d) No change of cage exploration assays under MeCP2 knock-down or SCT over-expression. *F*(2, 18) = 0.4858, *P* = 0.623. (e) MeCP2 knock-down deprived normal social affiliation behaviors in mice, and SCT over-expression in the BLA recovered social interaction levels. *F*(2, 18) = 17.87, *P* < 0.001. Tukey's post-hoc comparison: NC vs. KD, *P* = 0.016; KD + SCT vs. KD, *P* < 0.001. (f) Social preference ratio was impaired by MeCP2 deficiency and was rescued using SCT infection. *F*(2, 18) = 6.445, *P* < 0.008. Tukey's post-hoc comparison: NC vs. KD, *P* = 0.015; KD + SCT vs. KD, *P* = 0.001. *n* = 7 animals in each group in (d–f). (g) Heat maps reflecting normalized calcium transients (in Δ*F*/*F*_0_) of PrL neurons. A total of 100 representative neuronal soma were plotted for each group. (h) Total integrated calcium levels of PrL neurons were decreased by MeCP2 knock-down and were re-elevated under SCT over-expression in the BLA. Kruskal-Wallis test statistic = 132.6, *P* < 0.001. Dunn's multiple test, NC vs. KD, *P* < 0.001; KD + SCT vs. KD, *P* < 0.001. (i) The peak values of calcium transient were suppressed under MeCP2 knock-down and were potentiated by SCT over-expression. Kruskal-Wallis test statistic = 166.4, *P* < 0.001. Dunn's multiple test, NC vs. KD, *P* < 0.001; KD + SCT vs. KD, *P* < 0.001. (j) No remarkable change of calcium spike frequency (number of spikes) under either MeCP2 knock-down or SCT infection. Kruskal-Wallis test statistic = 5.417, *P* = 0.067. *n* = 120 neurons from 4 mice in each group in (h–j). (k) A cartoon illustration for the working model of SCT in social regulation. ns, no significant difference; *P < 0.05; **P < 0.01; ***P < 0.001. All data are presented as mean ± sem.

Lastly, we tested if these SCT-mediated social investigation behaviors can help to relieve relevant psychiatric disorders such as social dysfunctions in ASD. Different mouse ASD models converged to show synaptic defects in the prefrontal cortex [11,49], and we have proven the critical role of the BLA^SCT+^→mPFC pathway in social behaviors. We thus investigated the amygdala-cortical pathway in a mouse ASD model developed by ablating the expression of the methyl-CpG binding protein 2 (MeCP2) gene in the mPFC region (Fig. 8a and b, Fig. S14). We identified decreased social durations and preference ratio in our test arena (Fig. 8d–f), which agreed with previous studies showing the neuropathology and ASD-related social deficits that occurred upon MeCP2 mutation [50,51]. However, when the BLA region of these MeCP2 knock-down animals was infected with AAV-SCT to elevate local expression of SCT (Fig. 8c), impaired social behaviors were recovered (Fig. 8d–f). These results suggest that SCT may help to relieve social deficits. To provide a neural substrate for such behavioral modulations, we monitored the calcium activity of PrL cells using *in vivo* 2-photon microscopy (Fig. 8a). Results showed significantly reduced calcium activity under MeCP2 insufficiency, and re-potentiation by SCT over-expression in the BLA (Fig. 8g and h). In particular, SCT-mediated neuronal potentiation was mainly displayed by higher peak values of calcium spikes (Fig. 8i) rather than their frequencies (Fig. 8j), indicating stronger excitatory inputs. Such results were consistent with our findings showing the mono-synaptic excitatory connection between BLA^SCT+^ and mPFC cells (Fig. 5). In sum, our molecular, physiological, anatomical and behavioral evidence collectively identified two mutually exclusive BLA subpopulations, of which BLA^SCT+^ mediates social investigation behaviors via their mPFC projection, and BLA^SCT−^ cells are mainly anxiogenic neurons that can antagonize social interest (Fig. 8k).

## DISCUSSION

Our study defined a subpopulation of BLA neurons that can be activated upon social cues and elicit social interactions. These BLA^SCT+^ neurons might at least partially overlap with the ‘social ensemble’ [27] due to their similar percentage of total BLA neurons (∼30%) and their active role in social interaction behaviors. With distinct molecular and transcriptomic profiles, BLA^SCT+^ neurons preferentially project to the mPFC to facilitate social engagement, providing a bottom-up model in which social cues are integrated in the BLA to drive social behaviors in cortical regions. Further elaboration also revealed the activation of such a circuit by SCT in both naïve and ASD model mice, implying the potential value for clinical intervention when treating social deficits.

It has been suggested that amygdala neural circuits are involved in social behaviors [19], but are often correlated with negative valence coding [24] to suppress social engagement via the projection to midbrain nuclei such as the nucleus accumbens (NAc) [21,25]. Moreover, the top-down regulation from the prefrontal cortex towards amygdala nuclei affects social decision making [19,24], and GABAergic neurons in the MeA were recently reported to encode social reward via hypothalamic projections [22]. With regard to examining the positive control of control behaviors by BLA nuclei, the idea of amygdala representation of social exploration has recently been supported by the existence of the social ensemble in the BLA [27]. In juvenile mice, social approach was found to acutely activate BLA neurons [52], and our findings further identified these social-activated BLA neurons as the SCT+ subpopulation. Moreover, the one-cage social exploration assay used in the current study may carry confounding factors related to anxiety status, as the novel mouse was placed in the center of the field. We thus performed the classical 3-chamber assay, which replicated key findings, as mice presented less social interest when SCT was knocked down in the BLA, or when BLA^SCT+^ neurons were deactivated. These observations largely exclude the effect from anxiety-like behavior and demonstrated that this BLA^SCT+^ neural ensemble specifically regulates social interest toward same-sex novel mice.

Classical views claimed that the BLA mainly consisted of CaMKIIα+ excitatory cells and PV+ inhibitory neurons [47,53]. However, such a divergent system seems to be over-simplified as a recent study reported the unique role of CCK+ cells of the BLA in mediating depressive-like behaviors [30]. SCT was also found to be expressed in the BLA in our study and participated in a different behavioral module. While the majority of identified BLA subpopulations encode negative valence such as anxiety or fear, our BLA^SCT+^ cells are involved in processing positive stimulus. Indeed, recent studies are suggesting the existence of certain ‘reward-biased’ cells in the BLA and their projections to the PrL [54], and a molecular dissection study has partially revealed two distinct groups of BLA cells to provide a neural substrate of positive and negative valence, respectively [45]. Our study, after excluding the possible confounding effect of place preference, supports the coding of social-related positive valence by BLA^SCT+^ cells. In future, the presynaptic input of those neurons can be further studied to better dissect both internal and environmental factors during social sensation and initiation.

The BLA^SCT+^→mPFC pathway was shown here to affect social investigation behaviors. Currently available evidence suggests that the activation of the BLA→mPFC pathway suppresses social interaction in a resident-intruder test [24], but probably due to the concurrent anxiogenic effect [26]. Our study reported seemingly contradictory effects but can be explained by selectively manipulating BLA^SCT+^ cells, which consisted of <50% of the total number of mPFC-projecting cells. The existence of the BLA^SCT−^→mPFC pathway, plus our findings, which reveal the inhibition of social engagement upon BLA^SCT−^ cell activation, suggest that paralleled pathways may exist from the BLA to mPFC to suppress social engagement. When we examined the BLA^SCT+^→mPFC pathway, circuitry tracing and electrophysiological recording implied the excitatory nature of this synaptic connection. Moreover, *in vivo* calcium imaging of mPFC pyramidal neurons revealed the excitation of cortical neurons after BLA^SCT+^ cell activation, forming a bottom-up regulatory pathway. In the cortical region, since enhanced mPFC activity was closely correlated with social motivation and interaction [6,55,56], the BLA^SCT+^→mPFC circuit thus provides an excitatory input into the cortical region for mediating social behaviors.

The presynaptic input for BLA^SCT+^ cells has not been studied yet but should be interesting for further exploration. Based on currently available anatomic studies, a lot of BLA inputs came from the frontal cortex, such as the cingulate cortex [57] and prefrontal cortex [58]. In terms of behavioral modulations, the mPFC inputs into the BLA are mainly involved in anxiety-like behaviors. Therefore, mPFC projection to the BLA may provide a top-down information flow reflecting mental status for affecting the social outcome, as suggested by other studies highlighting the PFC-driven activation of amygdala-cortical neurons [59]. Recently, a whole-brain study reported that BLA cells receive inputs from the entorhinal cortex, auditory thalamus and auditory cortex [60]. This anatomical evidence implies the possible role of BLA^SCT+^ cells as a hub for sensing multi-module environmental cues to initiate social behaviors, as supported by our *in vivo* calcium recording data (Fig. 2). Besides the anatomical study, BLA^SCT+^ neurons have relatively higher excitability, making those cells more flexible to environmental stimulus. In future, the examination of BLA^SCT+^ cell input may help to better illustrate the regulatory mechanism of social interest.

Besides synaptic transmissions, neuroendocrine factors represent an alternative regulatory network for behavioral modulation. Both the mPFC and amygdala nuclei are known to express receptors of various neuropeptides such as oxytocin and vasopressin [61,62], both of which are widely accepted brain-derived endocrine factors that affect social behavior. In the prefrontal region, oxytocin plays a role in female social interactions via activating a specific group of oxytocin-receptor-expressing interneurons [63], which form a synaptic connection with BLA nuclei to facilitate social recognition [64]. In the BLA, local infusion of oxytocin enhanced social-related cognitive functions [65]. Similar phenotypes can be found in vasopressin, which also facilitates human social behaviors [66]. Our study has identified the existence of SCTR in the BLA in agreement with previous reports [67]. The coexistence of SCT and SCTR in the BLA thus suggests a possible autocrine or paracrine mechanism, in which locally released SCT can activate adjacent BLA neurons via ligand-receptor binding and intracellular downstream pathways. This hypothesis can be supported by molecular evidence showing that BLA^SCT+^ cells were enriched in neuropeptide binding and downstream molecular signaling (Fig. S7). In particular, the higher excitability of BLA neurons upon SCT infusion implies a self-propagated model in which social-activated neurons release SCT to confer higher excitability of themselves and other cells. As the downstream target, endogenous SCT over-expression activates the BLA^SCT+^→mPFC pathway to facilitate social affiliation. Taken together, SCT works as one neuroendocrine factor in mediating the amygdala-prefrontal neural circuit during social interaction.

Our results also suggested the effectiveness of SCT in activating the amygdala-prefrontal circuit to relieve social deficits in ASD models, agreeing with previous studies involving oxytocin and vasopressin [66]. However, clinical application using SCT to remediate ASD-related social symptoms did not obtain a satisfactory outcome in most trials [38]. The divergence between animal studies and human practice can be explained by at least two reasons: firstly, intravenous administration of SCT may not achieve effective concentration inside the brain due to the existence of the blood-brain barrier. Intranasal application, or other biomaterials as drug vehicles, may help to improve delivery efficiency. Secondly, SCT effectiveness may be compromised by its relatively rapid turnover rate, with <5 min of half-life in blood [68]. The optimization of the drug delivery system was thus required to achieve a sustained release of SCT. Alternatively, the development of small molecules for allosteric modulation of SCTR may also work as a candidate for drug development to treat social deficits.

In sum, our results identified a BLA neuronal ensemble with distinct molecular, physiological and anatomic features, which can facilitate social investigation behaviors. Such evidence sheds new light on the BLA as one of the converging centers for both environmental stimuli and internal statuses. In addition, the functional relevance of this SCT-mediated amygdala-prefrontal circuit provides more possibilities in the clinical remediation of social deficits.

## SUMMARY OF METHODS

**Experimental animals:** Adult C57BL/6 mice were used, in conjunction with in-house-bred SCT-floxed, SCT-Cre and Ai9 transgenic mice. All animals were kept in a standard specific pathogen free (SPF) animal house with normal light cycle. All animal procedures were approved by the Jinan University Institutional Animal Care and Use Committee.

**Molecular assays:** qPCR and enzyme-linked immunosorbent assay (ELISA) were used to quantify target gene/protein expression following RNA or protein extraction from target brain regions. RNAScope was employed for cell identity, and single-cell-based RNA sequencing was adopted for comparing the transcriptomic profile between BLA engrams.

**Histological studies:** Immunofluorescent staining was used for revealing the spatial distribution of target proteins.

***In vivo* calcium recording:** For monitoring BLA activity in behaviors, fiber photometry was employed using an implanted optic fiber. To observe the cortical neural activity, 2-photon calcium imaging was performed.

**Neural circuitry studies:** For the anatomical dissection, an antero- or retrograde labeling viral vector was injected into the target brain region. The BLA was infected with optogenetic or chemogenetic receptors using viral-mediated vectors, following optical stimulus or ligand infusion.

**Behavioral assays:** Open field test, elevated plus-maze and fear conditioning were employed to describe the general phenotype of mice. Two different social interaction assays—one-cage social exploration assay and classical 3-chamber assay—were performed in conjunction with circuitry recording or manipulation.

**Statistical analysis:** All data were firstly tested for normal distribution. Those fitting a normal distribution were analyzed by parametric approaches, whilst other data were compared using non-parametric methods.

Detailed materials and methods are available in the Supplementary Data.

## Data availability

All data are available within the article and the supplementary information or can be made available by the corresponding author (zhangli@jnu.edu.cn) upon reasonable request.

## Supplementary Material

nwac179_Supplemental_Files
