# Supplementary material for: Amygdala neural ensemble mediates mouse social investigation behaviors
Source: Natl Sci Rev. 2022 Aug 30;10(1):nwac179. doi: 10.1093/nsr/nwac179 (PMC9952061; doi:10.1093/nsr/nwac179)
Supplement: nwac179_Supplemental_Files [file nwac179_supplemental_files.zip › NSR_MS-2021-1298.R2_SI.pdf]

Supplemental Information

Amygdala neural ensemble mediates mouse innate social behaviors

Ji-an Wei, Qing Han, Zhihua Luo, Linglin Liu, Jing Cui, Jiahui Tan, Billy K.C. Chow, Kwok-Fai So, Li Zhang*

*Correspondence: zhangli@jnu.edu.cn (L.Z.)

This file includes:

Materials and Methods

Table S1

Figures S1-S14

Materials and Methods

Experimental animals

All procedures were carried out in accordance with protocols approved by the Jinan University Institutional Animal Care and Use Committee. Adult male mice (C57BL/6, 6 weeks old) were used as wild-type animals. Three lines of transgenic mice were employed: (1) Sct-floxed mouse line was obtained from Billy K.C. Chow's lab (The University of Hong Kong); (2) The SCT-Cre knock-in mouse line was generated by inserting IRES-iCre recombinase sequences after the 4th exon of SCT coding region (Beijing Vital River Laboratory Animal Technology, China); (3) The R26-tdTomato (Ai9) mouse was purchased from JAX Lab (Stock #007909). A double transgenic mouse line to label SCT+ cells were obtained by crossing male SCT-Cre mice with female Ai9 mice.

All animals were housed in a SPF-grade animal facility with 12/12 hr light-dark cycle (lights on at 8 AM) and were provided with food and water *ad libitum*. Genotyping of transgenic animals utilized the following primers:

(1) Sct-floxed mouse line:

Llox5-F:5'-ATAAGCGGCCGCGATTTGAGTTTCGGTGCTGG-3';

Rlox5-F:5'-ATAAGCGGCCGCGAGTGCCACCTTGCCCTG-3';

Rlox3-R:5'-GTCGTCGACGGTTTGGGGAGCCAGTATCT-3'

(2) SCT-IRES-Cre mouse line:

WT-F:5'-CACTCAGACCCTACAGGACTGGTGA-3';

WT-R:5'-ACATCACACACCATGAGCTAAAAGA-3';

Mut-F:5'-TGCCGGGTCAGAAAGAATG-3'.

Behavioral procedures

All experiments were performed during the light phase. Behavioral data were analyzed by people who were blinded to the experimental group.

Open field test: The open field test was performed in an acrylic box (50cm length × 50cm width × 40cm height). The mouse was placed in one of the corners of the arena at the start of each session, and was allowed to explore the arena for 5 min being recorded using a camera suspended above the box connected to a computerized video tracking system (Ethovision XT). The arena was cleaned thoroughly between each trial.

(Juvenile) Social exploration test: Mice were firstly housed in a big cage (50cm length × 30cm width × 20cm height) with clean padding 24 hr before the test. On the test day, an empty cylindrical metal cage (9 cm diameter, 15 cm height) was placed in the center of the test arena. During phase 1, the mouse was placed into the big cage having an empty metal cage. In phase 2, a same-sex juvenile mouse (4 weeks old) was placed into the metal cage. In each phase, the test mouse was allowed to explore the arena for 5 min. A camera connected to a computerized video tracking system (TopScan) recorded the movement path under dim light. The time of the mouse spent in actively sniffing within 1 cm around the cage was identified as cage exploration and social interaction, in phase 1 and phase 2, respectively. The social preference index was calculated as the ratio of the cage exploration duration against the social interaction length.

Three chamber social behavior test: The testing apparatus consisted of a white rectangular plastic box (60cm length × 40cm width × 20cm height) divided into 3 equal compartments (20cm length × 40cm width ×20cm height each) by frosted plastic dividers with a 6 cm opening in the

middle for mice to move freely. Before the test, mice were allowed to habituate in the entire box for 3 minutes. Mice which were not biased to either side were tested further for social preference. The social test trial consisted of 6 minutes. During phase 1, the mouse was placed into the box with two empty cylindrical metal cages (9 cm width × 15cm height) on each side for 3 min. In phase 2, one of the side chambers was randomly selected as the social side and the other as the non-social side. In the social chamber, a same-sex juvenile mouse (4 weeks old) was placed under the metal cage. The nonsocial side consisted of the same empty cage. A camera connected to a computerized video tracking system (TopScan) recorded the movement path with dim light. The time of the mouse spent in sniffing was identified as cage exploration and social interaction, in social side and nonsocial side, respectively. The social preference index was calculated as the ratio of the total exploration duration against the social interaction time.

Stereotaxic injection

Mice were anesthetized with isoflurane and were placed on a standard stereotaxic instrument (RWD, China). The hair was shaved, and local sterilization was administrated. The scalp was incised to expose the skull. After removing the periosteum tissue, basolateral amygdala (BLA, AP: -1.45 mm; ML: ±3.27 mm; DV: -4.75 mm) was located by the stereotaxic instrument. The injection hole was made by the high-speed micro-drill (OmniDrill35, WPI, USA), and the virus was injected into the target region in 50 nl/min. When the injection was finished, the glass electrode was retained for 10 min before withdrawl. For medial prefrontal cortex (mPFC, AP: 2.80 mm; ML: ±0.20 mm; DV: -1.00 mm) injection, the glass electrode was inserted in 30° angle to avoid the damage of image site.

All behavior experiments were performed at 2~3 weeks after the injection, as specified in each experimental protocol. For the anterograde tracing, AAV2/9-EF1α-DIO-EGFP-T2A-TK was injected at day 0, and HSV-ΔTK-hUbC-tdTomato was injected 2 weeks later at the same location. All virus vectors were listed in Table S1:

Optic fiber implantation and optogenetic stimulation

After the recovery from stereotaxic surgery, mice were anesthetized by isoflurane and fixed on the stereotaxic stage. The scalp was cut to expose and an optic fiber with ceramic ferrule (diameter: 1.25mm, ThinkerTech, China; for BLA stimulation length: 4.5mm; for mPFC stimulation length: 1.0 mm) was implanted (BLA, AP: -1.45 mm; ML: ±3.27 mm; DV: -4.65 mm. mPFC, AP: 2.80 mm; ML: ±0.20 mm; DV: on the surface of cortex). Two micro bone screws (RWD, China) were inserted into the skull around the fiber, which was fixed by the dental cement. Mice were allowed to recover in their home cage for 1 week. Before the conduction of recording session, each mouse was habituated in the test arena with fibers inserted.

At the beginning of the recording session, the test mouse with implanted optic fiber was connected to a fiber-optic patch cord (2 m length, ThinkerTech, China) suspended above with rotary joint (FRJ_1x1_FC-FC, Doric Lenses) during the social interaction. The patch cord was connected to a 594 nm laser or a 473 nm laser generated by power source (Changchun New Industries Optoelectronics Technology, China), with all output commands given by a trigger (ThinkerTech, China). For SCT+ neuronal or terminal activation by ChR2, bilateral optic fibers connected to the optic patch cords received 10-mW blue light in high frequency (20 Hz, 5 ms

pulses). For inhibition assay using NpHR3.0, a constant 10-mW yellow light was applied. For the ChrimsonR-mediated activation, a high-frequency (20 Hz, 5 ms pulses) yellow light was used.

For the social exploration test, the protocol was changed as a 3-phase version lasted for 6 min, which every phase lasted 2 min. From beginning to end, the metal cage and the same-sex juvenile mouse inside were placed in the center of the test arena. After a 5-min habituation, the laser was turned off during the first and third phase was off, and was turned on during the second phase.

For the 3-chamber social behavior test, the protocol was changed as a 4-phase version lasted for 12 min, which every phase lasted 3 min. A same-sex juvenile mouse (4 weeks old) was put into the metal cage on the random social side. The laser was turned off during the first and third phase and was turned on during the second and the fourth phase.

***In vivo* fiber photometry recording and data analysis**

The optic fiber was implanted as those of stimulating fiber, except that a recoding fiber with ceramic ferrule (diameter: 2.5mm, ThinkerTech, China) was used instead. A 3-colored fiber photometry apparatus (ThinkerTech, China) was used to record the neural activity of BLA^{SCT+} neuron in mice when encountering social cue. To record the fluorescence signals, excitation light was provided by LED sources (405nm Lumileds, 470 nm LUXEON Rebel, 572nm LUXEON Rebel) and was reflected by a dichroic mirror (Edmund Optics), focused through an objective lens (20x, NA=0.4, Olympus). An optical fiber (200nm OD, NA=0.37) guided the light between the commutator and the implanted optical fiber. The laser power was adjusted to the level of 40-60 μW at the input side. The fluorescence emission was filtered (87753, Edmund optics) and detected by a photomultiplier tube (H10721, Hamamatsu). An amplifier was used to convert the

photomultiplier tube current to the current voltage, which was further filtered through a low-pass filter (35Hz cut-off, ThinkerTech). The analog voltage signals were digitalized at 100Hz and recorded by fiber photometry software (ThinkerTech, China) based on Matlab package.

At the beginning of social cue exposure experiment, the value of calcium signal was recorded as F_{offset} when the fiber was not detached from the mouse head. The mouse with fiber connected was introduced into the test cage, and calcium signals were recorded by fiber photometry apparatus. The test cage was the same as of social interaction assay, with a round cage in the center of cage and a sniffing zone around the cage. When the nose of mouse approached or entered the sniffing zone, a tag was added on the recording trace automatically to set a sniffing initiation time point. Each session lasted for 5 min, during which 8-10 sniffing events usually occurred.

The recording data of fiber photometry were exported to analysis software (ThinkerTech, China). All raw data were firstly processed by the algorithm for correcting the decline of raw calcium signals caused by fluorophore protein bleaching in the recording process, and then were corrected against the reference channel (405nm). Event traces were extracted with reference to the tag (-2 s to +10 s relative to the sniffing initiation). The fluorescence change ($\Delta F/F_0$) values were calculated as $(F-F_0)/F_0-F_{offset}$, where F_0 is the baseline fluorescence signal averaged over a 2-sec control time window before each event triggering. The relative fluorescence values were eventually presented as heatmap series and plotted by average values with a shaded area indicating the standard errors of means (sem).

In vivo 2-photon calcium imaging and data analysis

Two-photon imaging on head-fixed, awake mice was performed at 3 weeks after the AAV2/9-CaMKIIa-GCaMP6s virus injection into mPFC. Mice were anesthetized with isoflurane, and the scalp was incised to expose the skull. After focal sterilization with 75% alcohol, the periosteum tissue over the skull surface was removed. A customized metal ring (8x8 mm) flanked by 2 metal bars was attached to the target skull with glue (Loctite 401, USA). The dental cement was applied around the ring to fix it on the skull. After the surgery, mice were allowed to recover overnight. Before imaging, mice received three 20-minute habituation under the head-fixed condition.

On the next day, an imaging window was made by the high-speed microdrill. Medical sponge was used to absorb excess bleeding followed by aCSF rinsing. A customized circle coverslip (diameter: 2.3mm) was placed on the cortical tissue surface and was sealed by Tissue Adhesive (3M, USA). The imaging experiment was performed on 920 nm excitation laser with a water-immersed objective (20×, 1.1 NA; ZEISS, Germany). The activity of neurons at 400-500 μm depth of mPFC were recorded in 2 Hz for 150 s. A total of 5 regions of interest (ROIs, at 400x400 μm size) were recorded in each animal.

The video of calcium image was firstly corrected by TurboReg plugin of ImageJ (Bethesda,MD, US). The ROIs of image were manually selected by the ROI Manager plugin, and fluorescence intensity (F) was calculated by mean pixels. Neurons presenting overexposure or no calcium peak were excluded. All data were normalized against F_0, which is the 5-sec mean fluorescence intensity of the lowest fluorescence value over 150s, and the relative value was calculated as $\Delta F/F_0=(F- F_0)/ F_0 \times 100\%$. The total integrated calcium value of a single neuron can be referred as the total output activity of neuron during the 150s imaging window. The frequency was calculated as the spike number per minute, and only the peak values over at least 2 standard deviations (SD)

of the mean value was identified as one valid signal. The amplitude of one neuron was the average peak value of all validated spikes during the imaging session.

***Ex vivo* electrophysiological recording**

Mice were deeply anesthetized with isoflurane and decapitated, and the brains were quickly removed and coronal slices (250 μm) containing the BLA were prepared by VT1000S Vibratome (Leica Microsystems, Wetzlar, Germany) in ice-cold, oxygenated (95% O_2 and 5% CO_2) artificial cerebrospinal fluid (aCSF, in mM: 126 NaCl; 2.5 KCl, 1.2 NaH_2PO_4, 10 Glucose, 26 $NaHCO_3$, 2.4 $CaCl_2$ and 1.2 $MgCl_2$, and 295 mOsm, at pH 7.4). The slices were recovered in warmed aCSF (33. 5 °C) for 30 min and were moved to room temperature for 30 min incubation before recording. Recording electrodes were prepared from filamented borosilicate glass capillary tubes (inner diameter, 0.86 μm) using a horizontal pipette puller (P-97; Sutter Instrument Co., Novato, CA). The pipette with resistance ranged from 4 to 6 MΩ were filled with intracellular solution containing (in mM): 135 K-gluconate, 5 KCl, 10 HEPES, 0.2 EGTA, 4 MgATP, 10 Na_2-phosphocreatine and 0.3 Na_3GTP, pH was adjusted to 7.4 with KOH.

To measure the effect of SCT on BLA neurons, a depolarizing current (0.8 s duration at 240 pA) was applied before perfusion, and the number of activated action potentials was calculated against the baseline, followed by 400 nM secretin perfusion in aCSF for 10 minutes. The number of action potentials was recorded when the membrane potential was held at -70 mV. To compare the electrophysiological features between SCT- and SCT+ neurons, graded depolarizing currents (from -90 pA to +300 pA, steps: 30 pA) were injected into both types of neurons, and the number of evoked action potentials was recorded when the membrane potential was held at -70 mV. To

validate the infection efficiency of ChR2, eNPHR3.0, ChrimsonR, hM4Di and hM3Dq, whole-cell current clamp was used to record infected neurons. The evoked action potential of ChR2 was measured under blue light pulses (1-20 Hz, 5 ms pulse duration). Evoked inhibition of action potentials of eNPHR3.0 was elicted by yellow light stimulation (1s duration). The spike responses of ChrimsonR-expressing neurons were induced with yellow light pulses (1-20 Hz, 5 ms pulse duration). For neurons expressing hM4Di or hM3Dq, evoked action potentials were recorded before and after perfusion with CNO (10 μM, 10 min). To characterize the BLA^{SCT+}-mPFC synaptic connection, evoked postsynaptic currents were induced by 5 ms blue light stimulation at axonal terminals of mPFC-projecting neurons infected with ChR2. To validate the monosynaptic connection, brain slices were perfused with tetrodotoxin (TTX ,1 μM) and 4-aminopyridine (4-AP, 100 μM) and NBQX (10 μM).

All data were sampled at 10 kHz and traces were filtered at 2 kHz. All recordings were performed using a Multiclamp 700B amplifier (Molecular Devices), and light pulses are transmitted through digital commands from Digidata1550 A. Series resistance (Rs) was maintained in the range of 10-20 MΩ and monitored throughout the experiments. If Rs changed >20% during recording, the data were excluded. Offline data analysis was performed using Clampfit 10.0 software (Molecular Devices).

Single cell sequencing by SMART-Seq

Before experiment, the operations area was decontaminated by RNaseZap (Sigma, USA). The somatic compartment of 15 SCT+ cells and 15 SCT- cells were aspirated by glass pipettes, and were separately ejected into a centrifuge tube containing the lysis buffer (SMART-Seq v4,

R400752, Clontech Laboratories, Inc.). The collected cell content was processed under *in vitro* reverse transcription to synthesize cDNA for further amplification. A modified oligo (dT) primer (the SMART CDS Primer) directed the 1st strand synthesis using SMARTScribe™ reverse transcriptase. When the synthesis reached the 5' end of the template, the enzyme's terminal transferase activity adds a few additional nucleotides to the 3' end of the newly formed cDNA. Using a second primer (SMARTer Oligonucleotide) which was base-paired with the 3' end of the cDNA, elongation was continued to obtain the full-length, single-stranded (ss) cDNA containing the adapter sequence on both ends. Using the primers targeting the adapter sequences at both ends, double stranded (ds) cDNA was amplified for cDNA library preparation.

The library construction begins with fragmented cDNA, which was generated by dsDNA fragmentase (NEB, M0348S) at 37°C incubation for 30min. Blunt-end DNA fragments were filtered by magnetic beads to select appropriate lengths (150~300 bp). Using a combination of fill-in reactions and exonuclease activity, a ligation reaction was performed to the adapter sequence as attached in cDNA synthesis. Using the ligated product as the template, further PCR amplification was performed to obtain the final library. The paired-end sequencing on an Illumina Novaseq™ 6000 at the (LC Sceiences,USA) following the vendor's recommended protocol, with 2*150 bp sequence length.

A cDNA library constructed by technology from the pooled RNA from brain samples of mouse was sequenced run with Illumina 4000 sequence platform. Using the Illumina paired-end RNA-seq approach, we sequenced the transcriptome, generating a total of million paired-end reads of bp length. Prior to assembly, the low-quality reads (containing sequencing adaptors, sequencing primer, or with quality score lower than 20) were removed. We next aligned reads of sample SCT+

cells and SCT- cells to the Ensembl (ftp://ftp.ensembl.org/pub/release-101/fasta/mus_musculus/dna/) *Mus muculas* reference genome using HISAT package, which initially remove a portion of the reads based on quality information accompanying each read and then maps the reads to the reference genome. HISAT allows multiple alignments per read (up to 20 by default) and a maximum of two mismatches when mapping the reads to the reference.

The mapped reads of each sample were assembled using StringTie. Then, all transcriptomes from samples were merged to reconstruct a comprehensive transcriptome using perl scripts. After the final transcriptome was generated, StringTie and edgeR was used to estimate the expression levels of all transcripts. StringTie was used to describe the expression level for mRNAs by calculating FPKM. The differentially expressed and genes were screened with log2 (fold change) >1 or <-1, plus a statistical significance (p value < 0.05) by R package.

Immunofluorescent staining

Animals were anesthetized with isoflurane and were perfused intracardially with 0.9% saline followed by 4% paraformaldehyde (PFA) in PBS. The whole brain was extracted and post-fixed in 4% PFA overnight at 4 °C, followed by transferring to a 30% PBS-buffered sucrose solution for 36 h at 4°C. Brains were sectioned into 40 μm-thickness coronal sections by a sliding microtome (Leica VT1000S). For the immunostaining, brain sections were washed in PBS for 5 times, and were blocked with CAS-Block (008120, ThermoFisher, USA) containing 0.3% Triton (T8787, Sigma-Aldrich, St Louis, MO) diluted in 0.01 M PBS. The primary antibody was added for 36 h incubation at 4°C. After washing with PBS and incubation with secondary antibody at room temperature for 2 h, sections were mounted on microscope slides (Citotest, China) and coverslips

were applied with Fluoro-Gel II with DAPI (EMS Catalog #17985-50). Antibodies used in this study were listed in Table S1.

Quantification of tissue SCT

After optogenetics stimulation of BLA^{SCT+} cells, the mouse was sacrificed after 15min. The whole brain was extracted and sectioned into thick slices. The BLA was separated from the section under a stereoscope (Zeiss, Germany). Total proteins were extracted with radioimmunoprecipitation assay (RIPA) lysis buffer (89900, ThermoFisher, USA). After protein quantification using the BCA Protein Assay Kit (P0011, Beyotime, China), the concentration SCT was measured by an ELISA kit (EK-067-04, Phoenix Pharmaceuticals, USA) following the manual instruction.

***In situ* hybridization using RNAScope approach**

The mouse was anesthetized with isoflurane and was perfused by PBS solution (RNAase-free). All sample preparation was based on formalin-fixed paraffin-embedded (FFPE) sample preparation and pretreatment protocols as recommended by RNAscope® Multiplex Fluorescent Assays v2 (Advanced Cell Diagnostics, USA). A customized SCTR probe (C1) was purchased from the Advanced Cell Diagnostics catalog. After removing the parafilm by xylene, Slides were washed in ethanol for 5 min. When slices were dry, hydrogen peroxide was used to remove hydrogen peroxidase of tissue. Proteins were digested using protease solution for 15min. Immediately, slides were washed twice in distilled water. In parallel, C1 and control probes were heated in a 40 °C water bath for 10 min. The probe was applied to the slides, which were covered by coverslips and placed in a 40 °C hybridization oven for 3 h. After washing for 3 times, slides underwent the

signal amplification process as stipulated in the vendor's protocols. Finally, slices were incubated with Opal 520 staining and were counter-stained by DAPI. The coverslip was added using ProLong Diamond Antifade mounting (ThermoFisher, USA).

Statistical analysis

All datasets were firstly tested for the normality before enrolling into statistical analysis. Those fitted normal distribution were analyzed by parametric approaches. In specific, two-sample *t*-test (paired or unpaired) was used to compare means between 2 groups. When more than 2 groups were compared together, one-way analysis of variance (ANOVA) was adopted, followed by Tukey post-hoc comparison between 2 specified groups. When two variables were simultaneously considered, two-way ANOVA was adopted. When dataset did not pass the normality test, nonparametric approaches were employed, including Mann-Whitney comparison between 2 groups, and Kruskal-Wallis test for multi-group comparison, in conjunction with Dunn's post-hoc comparison. The statistically significant level was defined when P value<0.05, and all data were presented as mean±sem.

Table S1 A list of chemicals and reagents used in this study.

**(A) Chemicals & peptides**		
**Name**	**Provider**	**Cat#**
SCT	Sangon Biotech	P17657
SCT5-27	Sangon Biotech	T510092
Avertin	Sigma-Aldrich, St Louis, MO	152463
Paraformaldehyde(PFA)	Sigma-Aldrich, St Louis, MO	158127
PBS	ThermoFisher, USA	10010023
CAS-Block	ThermoFisher, USA	008120
3% Triton	Sigma-Aldrich, St Louis, MO	T8787
Microscope slides	Citotest, China	80302-2101
Fluoro-Gel II with DAPI	EMS,USA	17985-50
Radioimmunoprecipitation assay(RIPA)	ThermoFisher, USA	89900
**(B) Antibodies**		
**Name**	**Provider**	**Cat#**
Rabbit anti CaMKIIα primary antibodies	Abcam,China	ab5683
Rabbit anti PV primary antibodies	Millipore, USA	MAB1572
Rabbit anti c-Fos primary antibodies	Cell Signaling Technology, USA	2250
Rabbit anti SCTR primary antibodies	Billy K.C. Chow's lab, HK	n/a
GFP primary antibodies	Abcam, China	ab13970
Dsred primary antibodies	Takara, Japan	632496
Goat anti-rabbit Alexa Fluor 488	Jackson ImmunoResearch, UK	111-545-003
Donkey anti-mouse Alexa Fluor 488	Jackson ImmunoResearch, UK	715-545-150
Donkey anti-rabbit Alexa Fluor 594	Jackson ImmunoResearch, UK	711-585-152
Donkey anti-chicken Alexa Fluor 594	Jackson ImmunoResearch, UK	703-545-155
**(C) Commercial assays**		
**Name**	**Provider**	**Cat#**
BCA Protein Assay Kit	Beyotime, China	P0011

SCT ELISA kit	Phoenix Pharmaceuticals,USA	EK-067-04
**(D) Viral vectors**		
**Name**	**Provider**	**Cat#**
AAV2/9-hSyn-Cre-EGFP-WPRE-pA	Taitool BioScience Co, Shanghai	S0230-9
AAV2/9-hSyn-EGFP-WPRE-pA	Taitool BioScience Co, Shanghai	S0237-9
AAV2/9-hSyn-DIO-GCaMP6m-WPRE-pA	Taitool BioScience Co, Shanghai	S0277-9
AAV2/9-CAG-DIO-taCaspase3-TEVp-WPRE-pA	Taitool BioScience Co, Shanghai	S0236-9
AAV2/9-Ef1α-DIO-hChR2(H134R)-mCherry-WPRE-pA	BrainVTA, Wuhan	PT-0002
AAV2/9-Ef1α-DIO-eNpHR3.0-EYFP-WPRE-pA	BrainVTA, Wuhan	PT-0006
AAV2/9-CaMKIIa-DO-hChR2(H134R)-EGFP-WPRE-hGH-pA	BrainVTA, Wuhan	PT-3315
AAV2/9-CaMKIIa-DO-eNpHR 3.0-EGFP-WPRE-pA	Taitool BioScience Co, Shanghai	S0465-9
AAV2/9-CaMKIIa-DO-ChrimsonR-EGFP-ER2-WPRE-pA	Taitool BioScience Co, Shanghai	S0462-9
AAV2/9-CaMKIIa-DO-EGFP-WPRE-hGH-pA	BrainVTA, Wuhan	PT-2108
AAV2/9-hSyn-DIO-mCherry-WPRE-pA	Taitool BioScience Co, Shanghai	S0240-9
AAV2/9-hSyn-DIO-hM4D(Gi)-mCherry-WPRE-pA	BrainVTA, Wuhan	PT-0020
AAV2/9-hSyn-DIO-hM3D(Gq)-mCherry-WPRE-pA	BrainVTA, Wuhan	PT-0019
AAV2/9-Ef1α-DIO-EGFP-2A-TK-WPRE-pA	BrainVTA, Wuhan	PT-0087
HSV-ΔTK-hUbC-tdTomato	BrainVTA, Wuhan	H03001
RV-N2C(G)-ΔG-eGFP	BrainVTA, Wuhan	R03001
AAV2/9-EF1a-secretin-P2A-mCherry-WPRE-hGH pA	BrainVTA, Wuhan	PT-2851
AAV2/9-U6-shRNA(scramble)-CMV-mCherry-pA	BrainVTA, Wuhan	PT-0923
AAV2/9-U6-shRNA3(Sctr)-CMV-mCherry-SV40 pA	BrainVTA, Wuhan	PT-3475
AAV2/9-U6-shRNA(mecp2)-hSyn-mCherry-WPRE-pA	Taitool BioScience Co, Shanghai	WZ023
AAV2/9-U6-shRNA(NC)-CAG-mCherry-pA	Taitool BioScience Co, Shanghai	S0346-9
AAV2/9-CaMKIIa-GCaMP6s-WPRE-pA	BrainVTA, Wuhan	PT-0110

AAV2/9-CaMKIIa-DIO-eNpHR3.0-mCherry-WPRE-hGH-pA	BrainVTA, Wuhan	PT-2060
AAV2/9-CaMKIIa-DIO-hChR2(H134R)-mCherry-WPRE-hGH-pA	BrainVTA, Wuhan	PT-2059
AAV2/9-CaMKIIa-DIO-GCaMp6s-WPRE-hGH-pA	BrainVTA, Wuhan	PT-0090

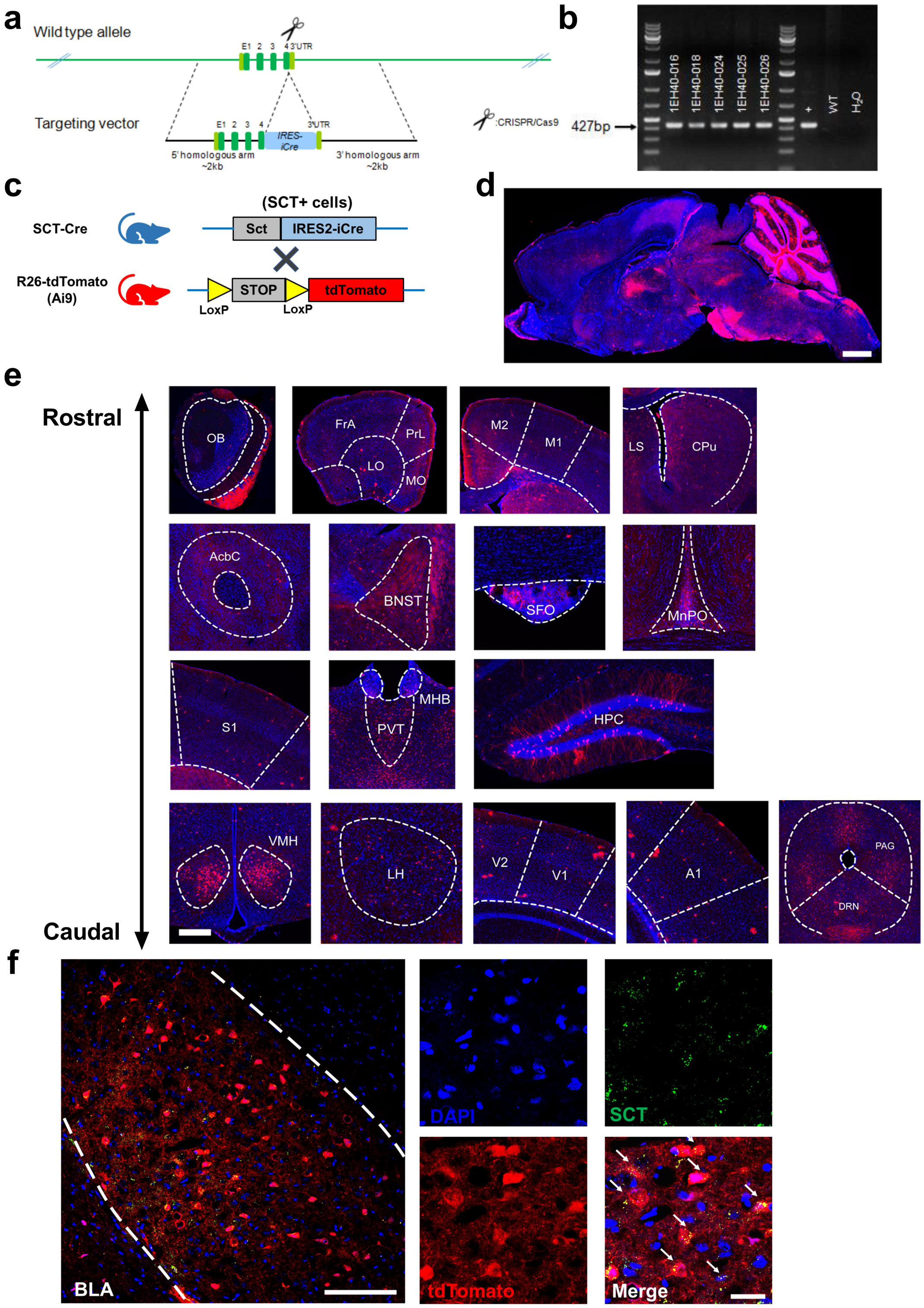

Figure S1. Generation and characterization of SCT-Cre knock-in mice. (a) Targeted gene inversion of IRES-iCre recombinase sequences after the 4th exon of SCT coding region. **(b)**

Genotyping of mouse tissues confirmed successful gene knock-in. **(c)** Schematic illustration for SCT-Cre;Ai9 double transgenic mice. An *in vivo* gene recombination occurs in SCT+ cells, in which Cre recombinase activated the expression of tdTomato as the fluorescent label. **(d)** A mid-sagittal fluorescent image of the whole brain of SCT-Cre;Ai9 mouse. Scale bar, 500 μm. **(e)** Sample images of different brain regions indicating the spatial distribution profile in SCT in the mouse brain. Scale bar, 150 μm. **(f)** RNA Scope of SCT transcript in the BLA of SCT-Cre;Ai9 double transgenic mice. Amounts of overlapping between SCT mRNA and tdTomato can be observed (as arrows indicated). Scale bars, 150 μm in the pan-BLA view, and 50 μm in the enlarged inserts.

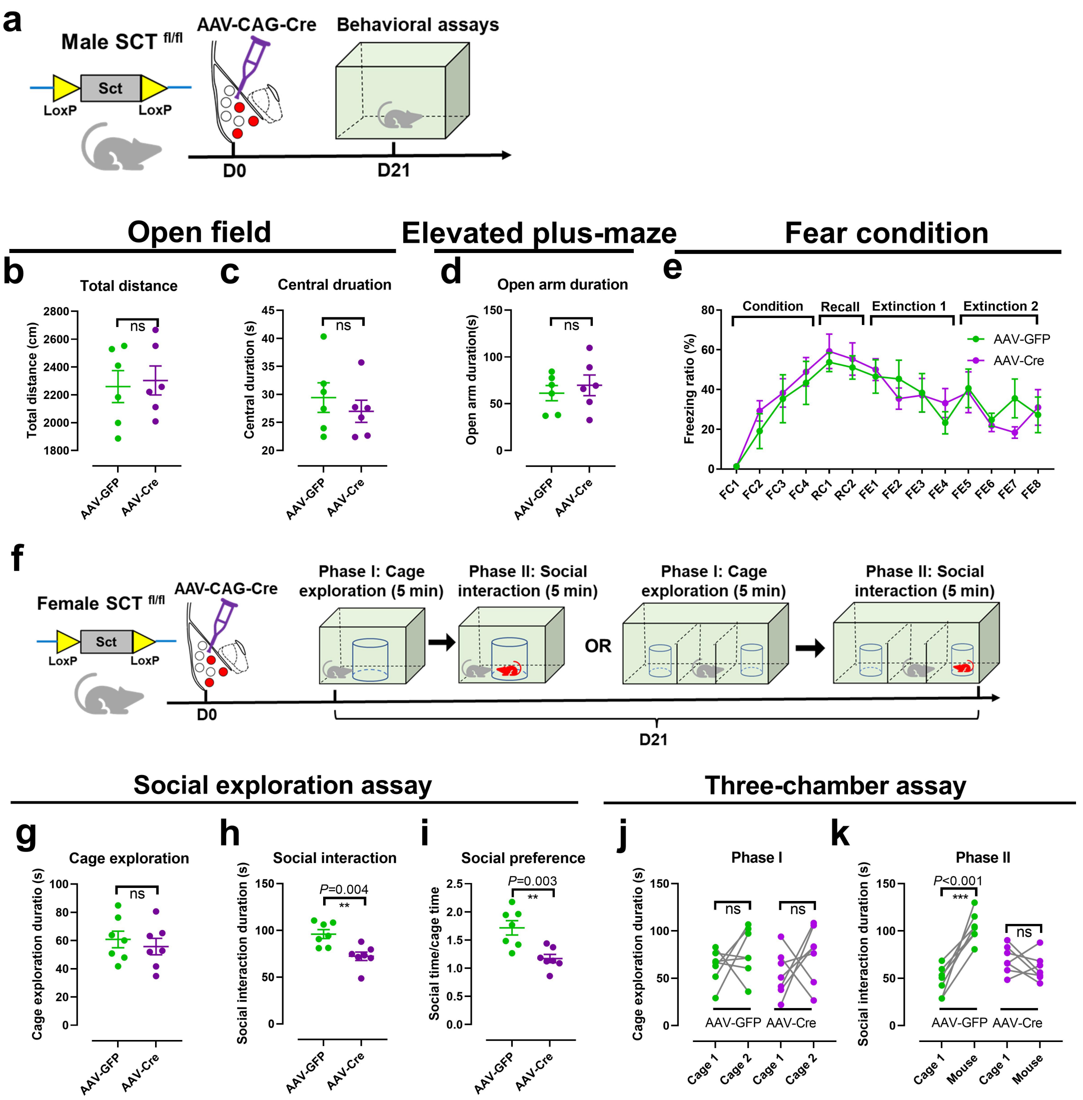

Figure S2. Behavioral phenotyping of SCT in male and female mice. (a) Schematic diagram of conditional knockout of SCT by injecting AAV-Cre vector into homozygous SCT-floxed mice. **(b)** Open field test showed no significant change in total distance. Two-sample unpaired *t*-test, *t*(10)=0.2818, *P*=0.784. **(c)** Similar central duration under SCT deprivation. *t*(10)=0.7400, *P*=0.476. **(d)** No change of open arm duration on the elevated plus-maze. *t*(10)=0.6089, *P*=0.556. **(e)** Unaltered acquisition, retrieval and extinction of fear conditioning memory by SCT knockdown in BLA. Two-way ANOVA respect to group factor, *F*(1, 120)=0.0855, *P*=0.771. *N*=6 mice in each group in **(b-e)**. **(f)** Experimental design of BLA-specific SCT gene knockdown assay.

Female homozygous SCT-floxed ($SCT^{fl/fl}$) mice received AAV-CAG-Cre injection into bilateral BLA region, followed by (juvenile) social exploration assay or 3-chamber assay 3 weeks later. Experimental design was the same as those in Figure 1f. **(g)** No significant change of cage exploration time (during phase I). $t(12)=0.6084$, $P=0.554$. **(h)** Decreased social interaction time after BLA-specific SCT gene knockdown. $t(12)=3.611$, $P=0.004$. **(i)** Lower social preference ratio (=social time/cage exploration time) in SCT knockdown group. $t(12)=3.742$, $P=0.004$. $N=7$ mice each group in **(g-i)**. **(j)** Both groups presented minor place preference when 2 empty cages were placed. Paired *t*-test, AAV-GFP group, $t(6)=1.007$, $P=0.353$; AAV-Cre group, $t(6)=1.066$, $P=0.328$. **(k)** During phase II when one novel mouse was placed, AAV-Cre mice ($t(6)=1.107$, $P=0.311$) lost social interest compared to AAV-GFP controls ($t(6)=6.555$, $P<0.001$). $N=7$ mice per group in **(g-k)**. ns, no significant difference; **, $P<0.01$; ***, $P<0.001$. All data were presented as mean±sem.

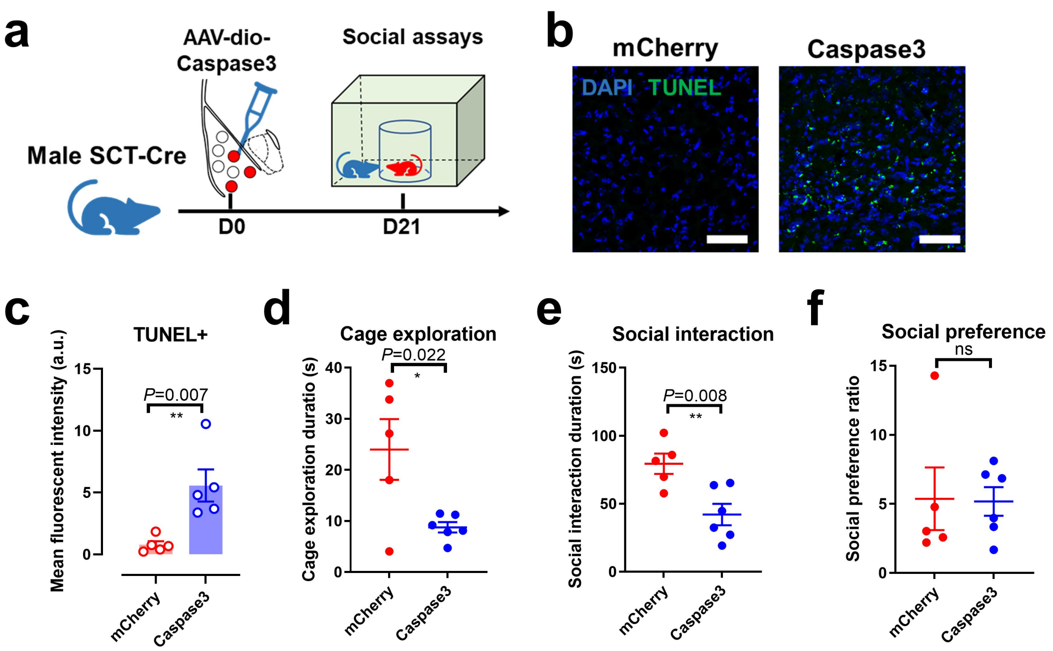

Figure S3. Ablation of BLA^{SCT+} cells impaired social interactions. (a) Schematic illustrations of experiments, in which an AAV-dio-Caspase3 was introduced into BLA of SCT-Cre mice. Three weeks later, social behaviors were evaluated. **(b)** Representative BLA slices for visualizing cell deaths using TdT-mediated dUTP Nick-End Labeling (TUNEL) approach. Scale bar, 100 μm. **(c)** Caspase3 transfection remarkably elevated programmed cell death level, as suggested by elevated TUNEL+ signal intensity. Two-sample unpaired *t*-test, $t(9)=3.602$, $P=0.007$. **(d)** Decreased cage exploration duration in Caspase3 transfected group. $t(9)=2.776$, $P=0.022$. **(e)** Shorter social sniffing durations after ablation of SCT+ cells. *t*-test, $t(9)=3.394$, $P=0.008$. **(f)** Unchanged social preference ratio between two groups. *t*-test, $t(9)=0.0803$, $P=0.938$. $N=5$ and 6 mice in mCherry and Caspase3 group, respectively, in **(c-f)**. ns, no significant difference; *, $P<0.05$; **, $P<0.01$. All data were presented as mean±sem.

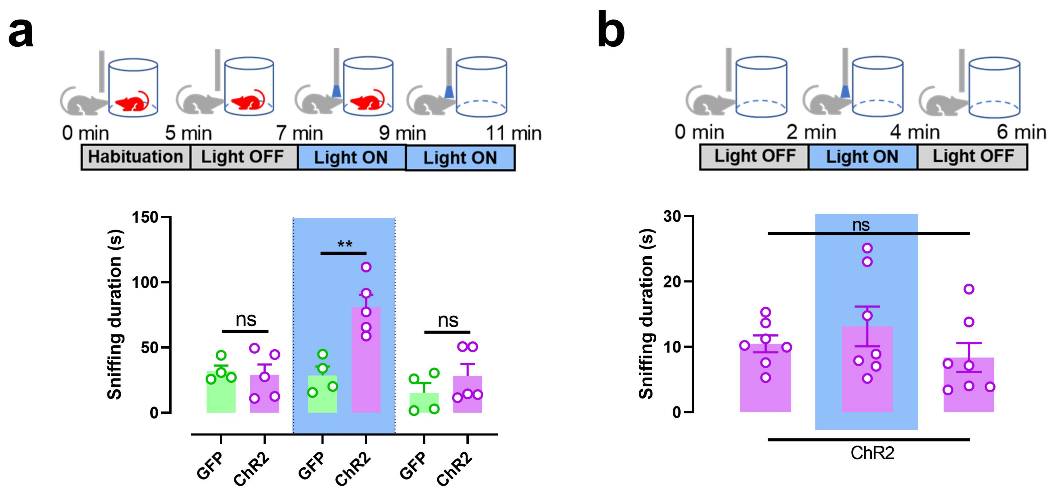

Figure S4. Irrelevance of BLA^{SCT+} cells with place preference. (a) Upper, schematic illustrations of social behavioral assays. SCT-Cre mice were transfected with ChR2, followed by social assays as in **Figure 3a**, except that in the last phase, the novel mouse was removed and the light stimulation persisted. Lower, quantification of social time reflected remarkably elevated social time upon ChR2, but only in the presence of target mouse. One-way ANOVA, $F(5, 21)=8.615$, $P<0.001$. Tukey's post-hoc comparison between GFP and ChR2: phase 1 (Light OFF), $P=0.998$; phase 2 (Light ON), $P=0.002$; phase 3 (Light ON without target mouse), $P=0.862$. $N=4$ and 5 mice in GFP and ChR2 group, respectively. **(b)** Upper, experimental procedures for the place preference assay. Light stimulation was applied as in **Figure 3a**, except that no target mouse was placed in the central cage. Lower, no preference toward the central cage. One-way ANOVA, $F(2, 18)=1.083$, $P=0.360$. $N=7$ mice. ns, no significant difference; **, $P<0.01$. All data were presented as mean±sem.

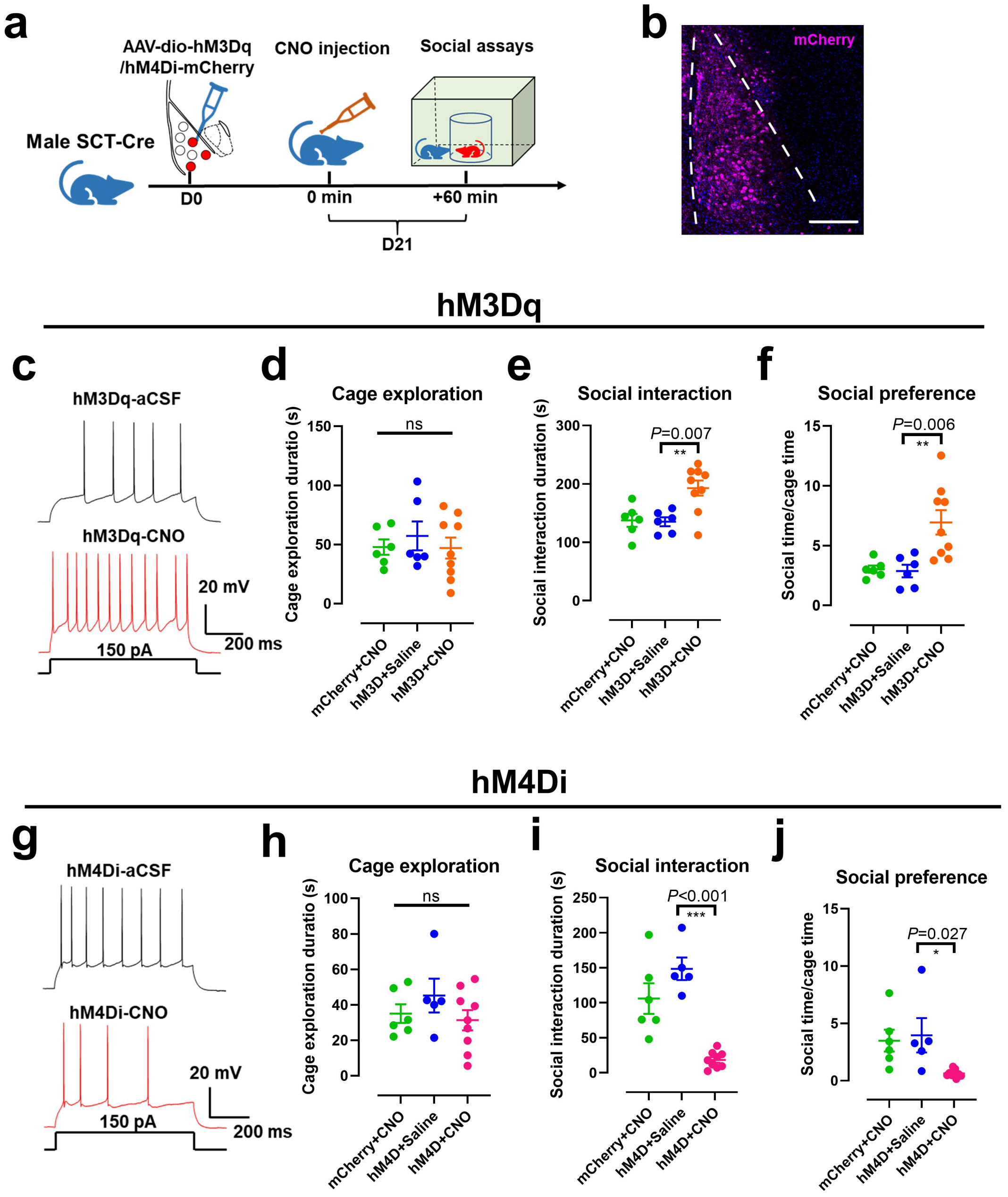

Figure S5. Chemogenetic manipulation of BLA^{SCT+} ensembles affected mouse social behaviors.

(a) Experimental protocols. Male SCT-Cre mice received stereotaxic injection of AAV-dio-hM3Dq/hM4Di into bilateral BLA. Three weeks later, the binding ligand clozapine-N-oxide (CNO) was intraperitoneally injected, followed by social assays in 30 min. **(b)** Infection sites of viral vectors. Scale bar, 200 μm. **(c)** Sample traces of membrane potentials from *ex vivo* brain slice recording after CNO application demonstrated effective neuronal activation. **(d)** No change of cage

exploration time upon CNO application. One-way ANOVA, $F(2, 18)=0.3337$, $P=0.721$. **(e)** Elevated social sniffing durations after CNO administration. One-way ANOVA, $F(2, 18)=8.353$, $P=0.003$. Tukey's post-hoc comparison, CNO vs Saline, $P=0.007$. **(f)** Potentiated social preference index upon CNO-induced neuronal activation. One-way ANOVA, $F(2, 18)=8.612$, $P=0.002$. Tukey's post-hoc comparison, CNO vs Saline, $P=0.006$. $N=6$, 6 and 9 mice in mCherry, Saline and CNO groups, respectively, in **(d-f)**. **(g)** Membrane potentials by chemogenetic inhibition of BLA^{SCT+} cells. **(h)** Unaltered cage exploration time. One-way ANOVA, $F(2, 17)=1.080$, $P=0.362$. **(i)** Suppressed social affiliation upon CNO treatment. One-way ANOVA, $F(2, 17)=25.43$, $P<0.001$. Tukey's post-hoc comparison, CNO vs Saline, $P<0.001$. **(j)** Decreased social preference ratio after CNO-induced neuronal inhibition. One-way ANOVA, $F(2, 17)=5.469$, $P=0.014$. Tukey's post-hoc comparison, CNO vs Saline, $P=0.027$. $N=6$, 5 and 9 mice in mCherry, Saline and CNO groups, respectively, in **(h-j)**. ns, no significant difference; *, $P<0.05$; **, $P<0.01$; ***, $P<0.001$. All data were presented as mean±sem.

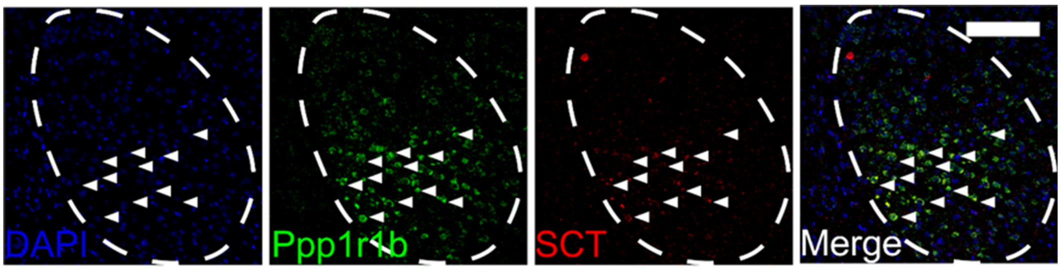

Figure S6. The overlap between SCT+ and Ppp1r1b+ cells in BLA. The RNA Scope assay for Ppp1r1b was performed on SCT-Cre;Ai9 reporter line, and found that SCT+ cells mostly belonged to a subpopulation of Ppp1r1b+ cells. Scale bars, 150 μm.

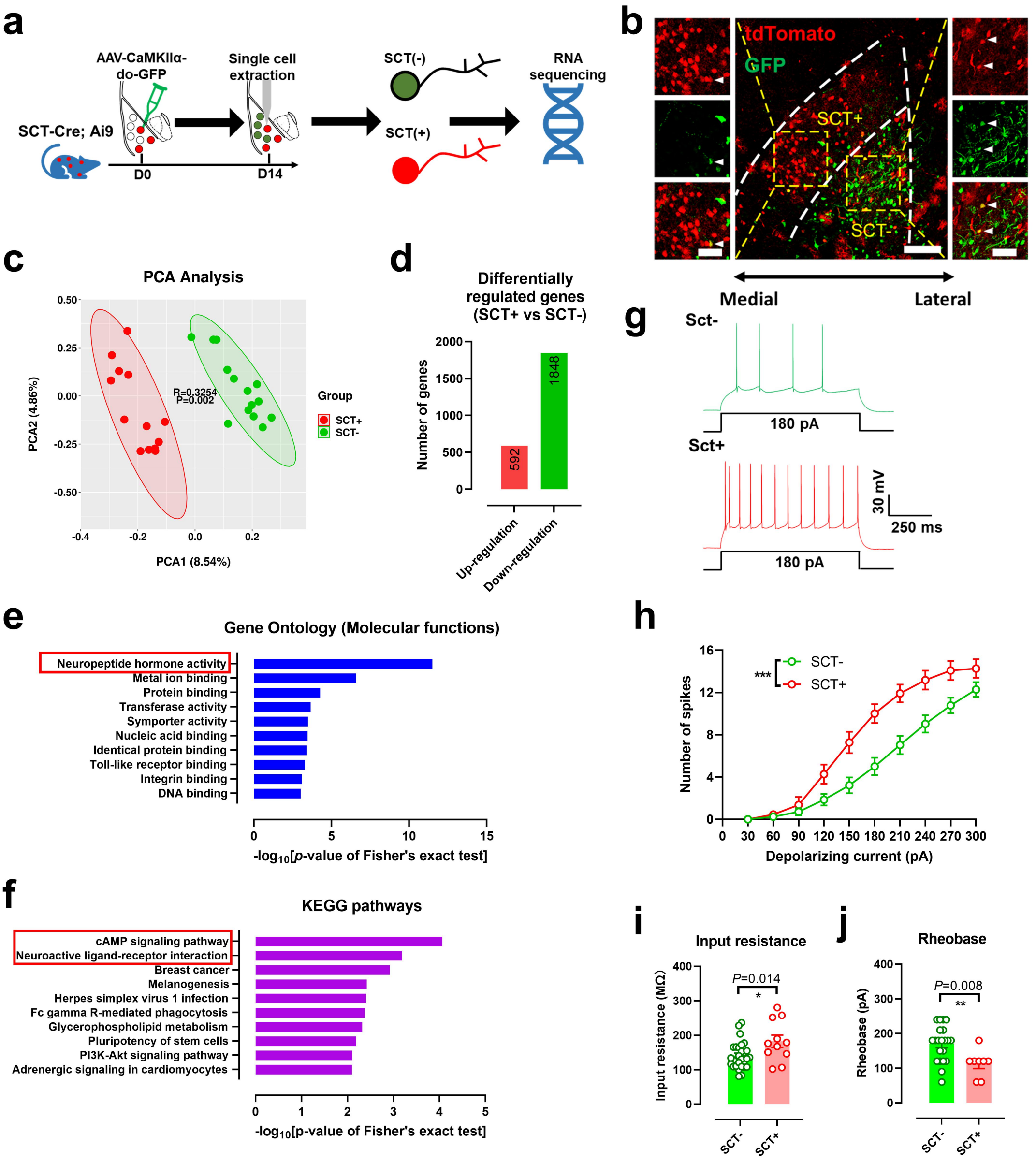

Figure S7 SCT defines two groups of BLA neurons with molecular and electrophysiological properties. (a) Schematic diagram of cell identification assays. SCT-Cre;Ai9 reporter mice received AAV-do-GFP injection to label SCT- cells. After 2 weeks, acute brain slices were prepared for patch clamp recording, followed by cell contents extraction and single-cell based transcriptomics studies. **(b)** Sample fluorescent images showing the distribution of SCT+ (tdTomato) and SCT- cells in BLA. Scale bars, 100 μm in the panoramic view (central), and 50 μm in the small inserts (left and right panels). **(c)** Principal component analysis of transcriptomics showed divergent profiles between

BLA^{SCT+} and BLA^{SCT-} cells. *n*=13 and 14 cells from 4 mice in SCT- and SCT+ group, respectively. **(d)** Number of differentially expressed genes (fold change > 2, and *P* value<0.05). **(e)** Gene Ontology enrichment of molecular functions for differentially expressed genes. **(f)** Kyoto Encyclopedia of Genes and Genomes (KEGG) analysis for molecular pathways of significantly regulated genes. **(g)** Sample traces of cell voltage recording when SCT- (upper) or SCT+ (lower) cells received an injection current of 180 pA. **(h)** Frequency of induced spiking of two cell sub-types, with gradually increased current injections. BLA^{SCT+} neurons displayed higher excitability as more spiking can be observed under the same stimulus. Two-way ANOVA with respect to current× group interaction effect, $F(9,333)=6.671$, $P<0.001$. **(i)** BLA^{SCT+} cells had higher input resistance compared to BLA^{SCT-} group. Two-sample unpaired *t*-test, $t(37)=2.591$, $P=0.014$. **(j)** BLA^{SCT+} cells presented lower rheobase values, suggesting higher excitability. Two-sample unpaired *t*-test, $t(37)=2.862$, $P=0.008$. *n*=28 and 11 neurons from 4 mice in SCT- and SCT+ group, respectively, in **(h-j)**. *, $P<0.05$; **, $P<0.01$; ***, $P<0.001$. All data were presented as mean±sem.

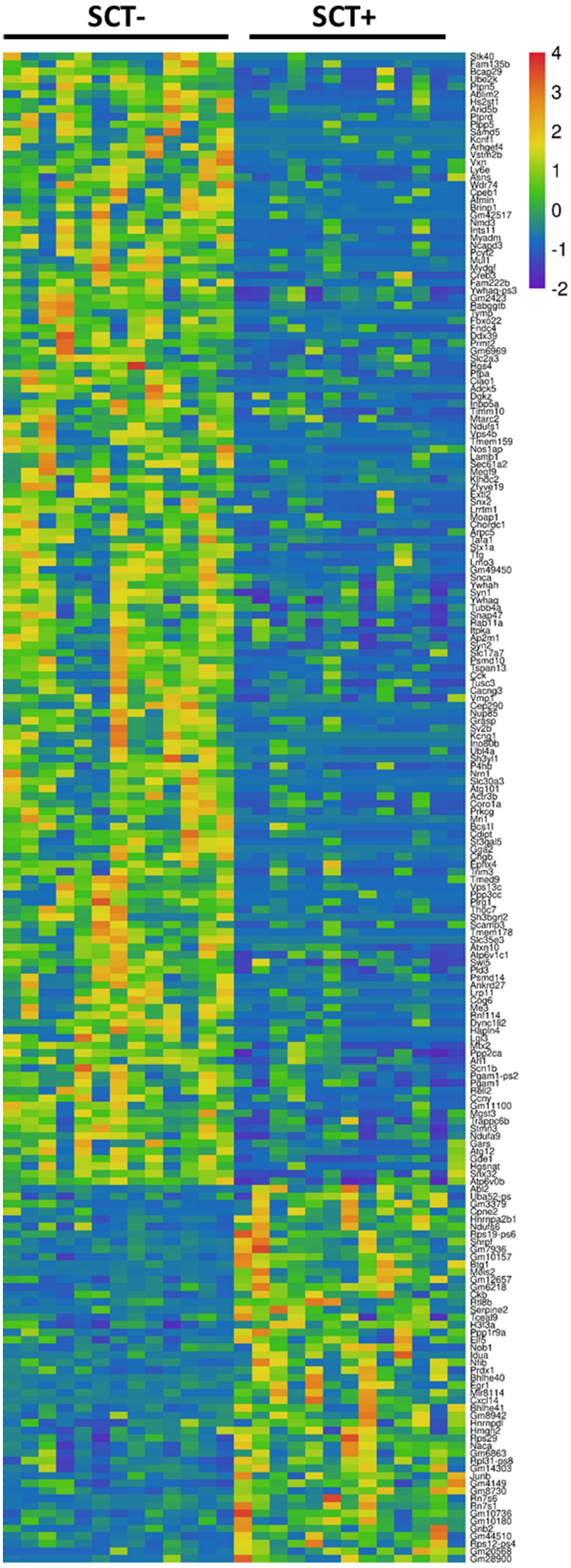
SCT-
SCT+
4
3
2
1
0
-1
-2

Figure S8. Differentially regulated genes between BLA^{SCT+} and BLA^{SCT-} cells. A total of 200 representative genes (with the most significantly difference) were plotted for each sample (neuron). Transcription levels (in FKPM) have been normalized as z-score, which was color-coded as the legend indicated. *n*=13 and 14 cells from 4 mice in SCT- and SCT+ group, respectively. For a full list of all differentially regulated genes, see Extended Data Table 1.

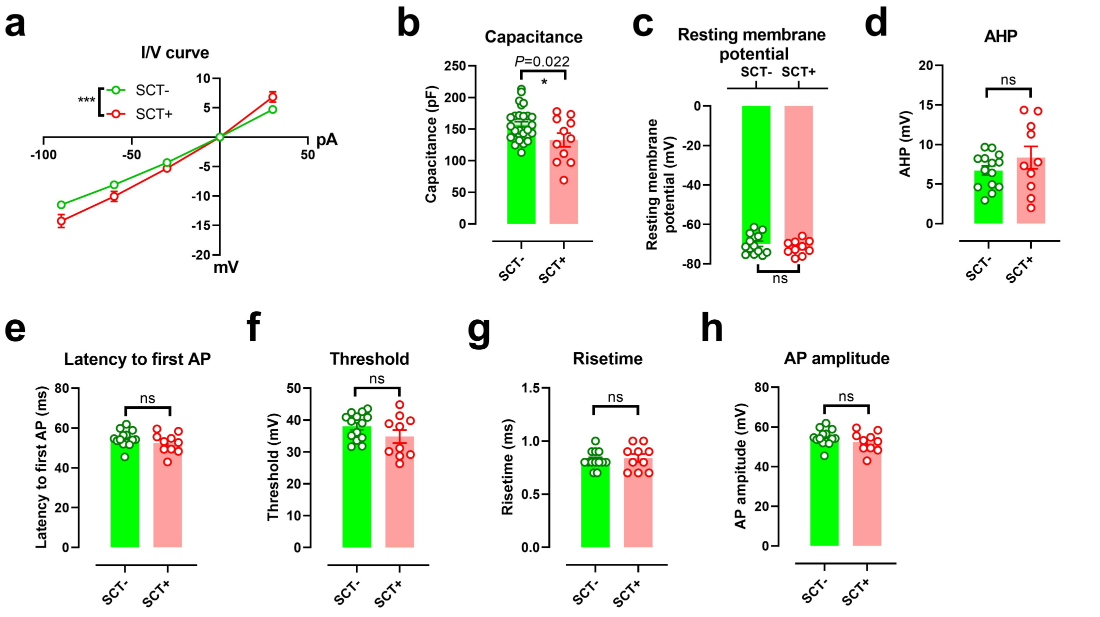

Figure S9. Altered membrane permeability but unchanged action potential (AP) kinetics between BLA^{SCT+} and BLA^{SCT-} cells. (a) An I/V curve showing higher curve slope in $BLA^{SCT=}$ cells, indicating change membrane permeability of ions. Two-way ANOVA with respect to group×stimulating current amplitude effect, $F(4, 185)=6.690$, $P<0.001$. **(b)** Decreased membrane capacitance in BLA^{SCT+} neurons. Two-sample unpaired *t*-test, $t(37)=2.390$, $P=0.022$. $n=28$ and 11 neurons from 4 mice in BLA^{SCT-} and BLA^{SCT+} group, respectively. **(c)** Unchanged resting membrane potential. $t(22)=0.9900$, $P=0.333$. **(d)** Similar after-hyperpolarization potential (AHP) levels. $t(22)=1.193$, $P=0.246$. **(e)** Unchanged latency to first AP among two types of BLA cells. $t(22)=1.229$, $P=0.232$. **(f)** Similar threshold of AP. $t(22)=1.495$, $P=0.149$. **(g)** Risetime of AP was largely unaffected. $t(22)=0.4052$, $P=0.690$. **(h)** Regular AP amplitudes. $t(22)=1.229$, $P=0.232$. $n=14$ and 10 neurons from 3 mice in BLA^{SCT-} and BLA^{SCT+} group, respectively, in **(c-h)**. ns, no significant difference; *, $P<0.05$; **, $P<0.01$; ***, $P<0.001$. All data were presented as mean±sem.

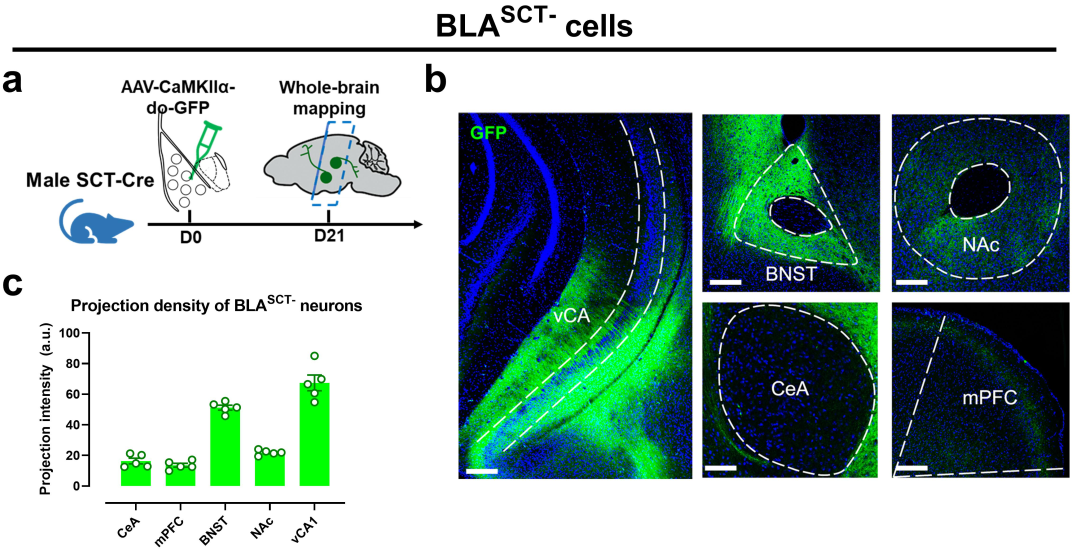

Figure S10. The projection pattern of BLA^{SCT-} neurons. (a) Experimental flowcharts for visualizing BLA^{SCT-} neuron projection sties. SCT-Cre mice were transfected with AAV-do-GFP to label BLA^{SCT-} cells, whose axonal terminus were examined across the brain. **(b)** Major projecting targets of BLA^{SCT-} cells. Scale bar, 100 μm. **(c)** Quantification of projecting density by calculating fluorescent intensity. CeA, central amygdala nuclei; mPFC, medial prefrontal cortex; BNST, bed nucleus of the stria terminalis; NAc, nucleus accumbens; vCA1, ventral CA1 of hippocampus. *N*=5 mice per group (averaged from 4 slices per animal).

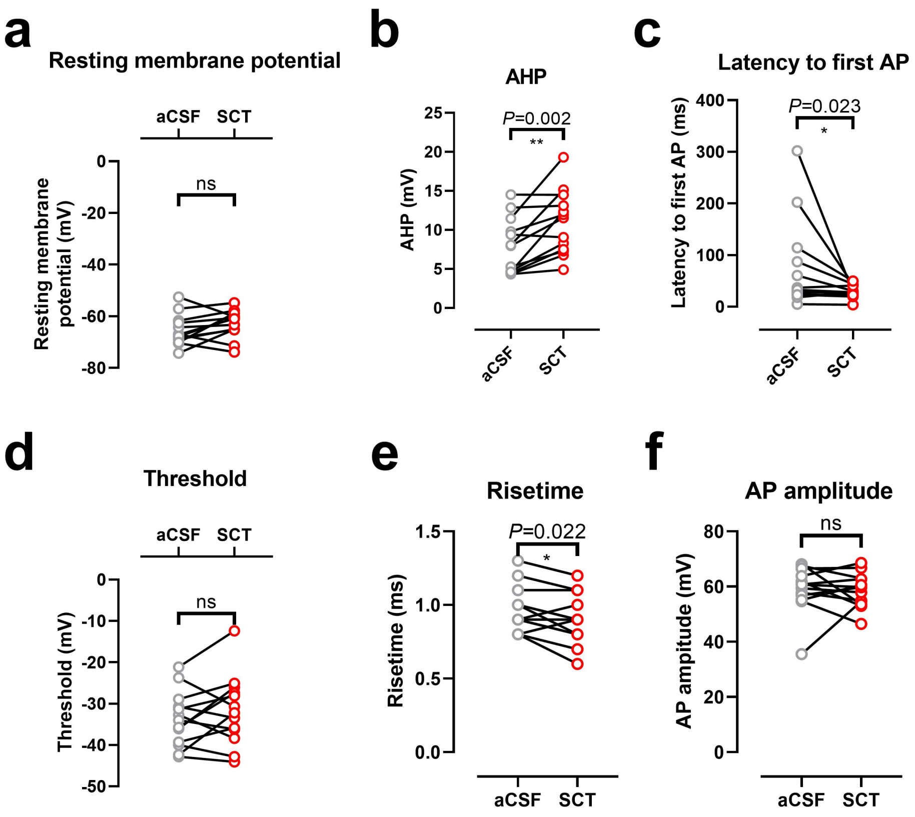

Figure S11. SCT potentiates the excitability of BLA neurons. (a) No change of resting membrane potential after SCT administration. Two-sample paired *t*-test, *t*(12)=1.779, *P*=0.101. **(b)** Higher AHP levels after introducing SCT *ex vivo*. *t*(12)=3.861, *P*=0.002. **(c)** Decreased latency to the first AP afterwards. *t*(12)=2.608, *P*=0.023. **(d)** Unchanged threshold for AP. *t*(12)=1.199, *P*=0.2538. **(e)** Shortened risetime of AP. *t*(12)=2.635, *P*=0.022. **(f)** No change of AP amplitude under SCT perfusion. *t*(12)=0.2357, *P*=0.818. *n*=13 neurons from 4 mice in **(a-f)**. ns, no significant difference; *, *P*<0.05; **, *P*<0.01. All data were presented as mean±sem.

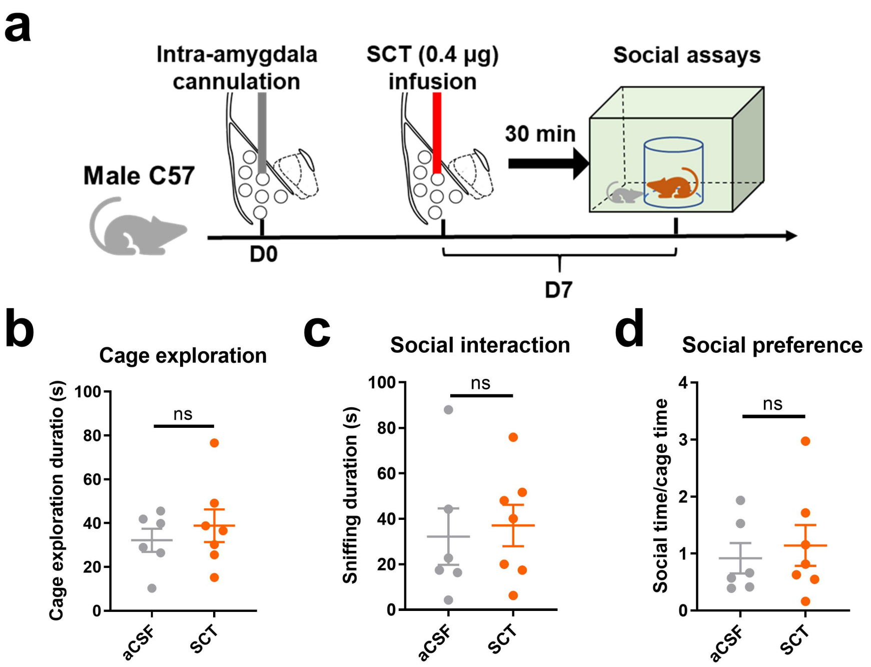

Figure S12. Unaffected social interaction under hostile conditions. (a) Schematic diagram of social assays, which adopted the same protocol as in **Figure 6g**, except that one aggressive CD1 mouse was placed in the central cage. **(b)** No change of cage exploration durations. Two-sample unpaired *t*-test, $t(11)=0.7036$, $P=0.496$. **(c)** Unaltered social sniffing durations. $t(11)=0.3242$, $P=0.752$. **(d)** Social preference towards a CD1 mouse was not affected by SCT infusion. $t(11)=0.4911$, $P=0.633$. $N=6$ and 7 mice in aCSF and SCT group, respectively, in **(b-d)**. ns, no significant difference. All data were presented as mean±sem.

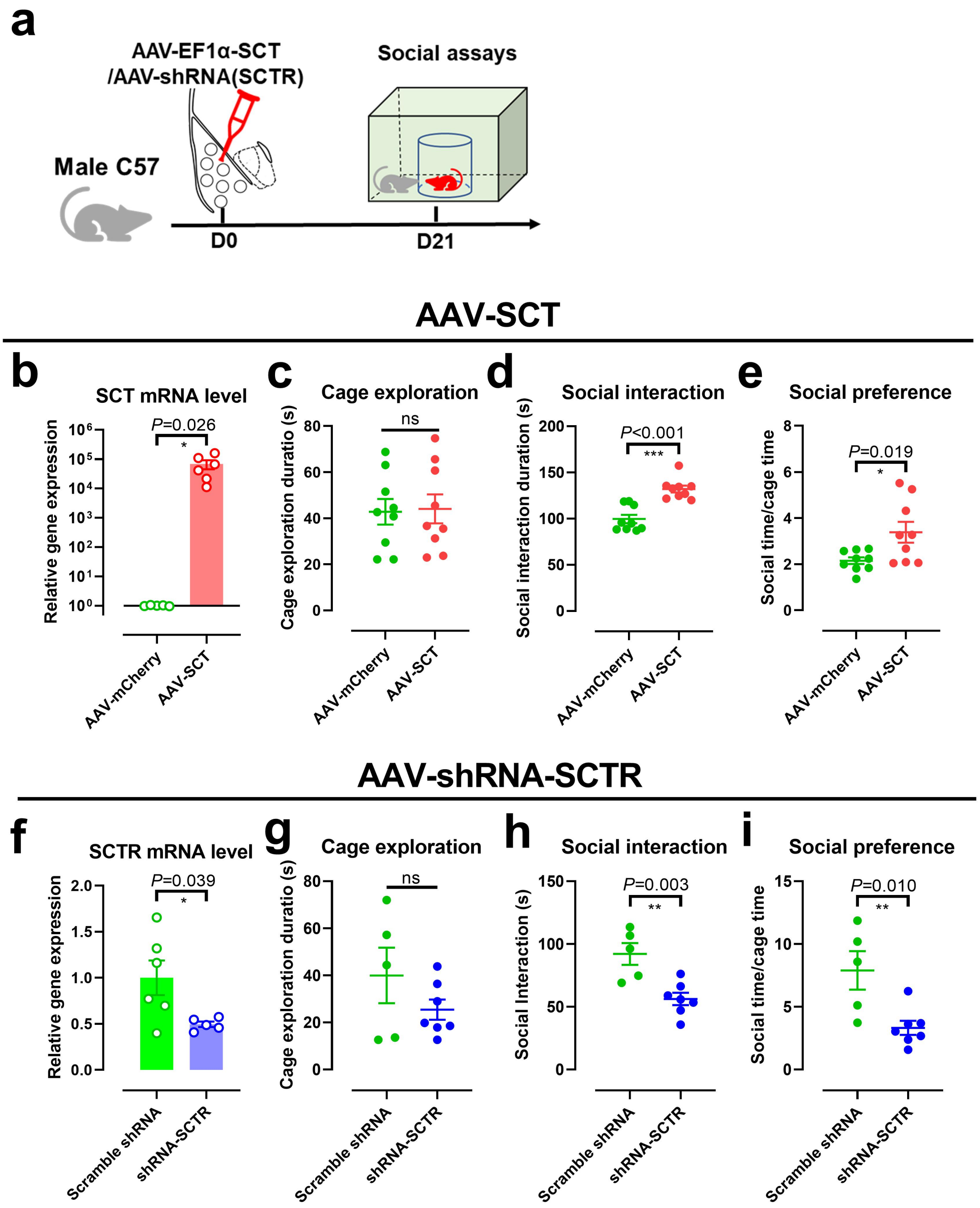

Figure S13. SCT-SCTR axis in BLA modulates social interaction. (a) Schematic diagram of experimental design. Social assays were performed at 3 weeks after stereotaxic injection of AAV-SCT or AAV-shRNA targeting SCTR. **(b)** AAV transfection remarkably increased endogenous SCT expression. Two-sample unpaired *t*-test, $t(9)=2.670$, $P=0.026$. $N=5$ and 6 mice in mCherry and SCT group, respectively. **(c)** No change of cage exploration time after SCT over-expression. $t(16)=0.1510$,

P=0.882. **(d)** Chronic SCT transfection social sniffing duration of wild type mice. $t(16)=5.503$, $P<0.001$. **(e)** Stronger social preference under SCT treatment, $t(16)=2.605$, $P=0.019$. $N=9$ mice in each group in **(c-e)**. **(f)** shRNA transfection effectively repressed SCTR expression. $t(9)=2.408$, $P=0.039$. $N=6$ and 5 mice in Scramble and shRNA-SCTR group, respectively. **(g)** SCTR knockdown did not affect cage exploration time. $t(10)=1.316$, $P=0.218$. **(h)** Decreased social interaction time after SCTR gene knockdown. $t(10)=3.851$, $P=0.003$. **(i)** Social preference ratio was decreased by SCTR knockdown. $t(10)=3.199$, $P=0.010$. $N=5$ and 7 mice in Scramble and shRNA-SCTR group, respectively, in **(f-i)**. ns, no significant difference; *, $P<0.05$; **, $P<0.01$; ***, $P<0.001$. All data were presented as mean±sem.

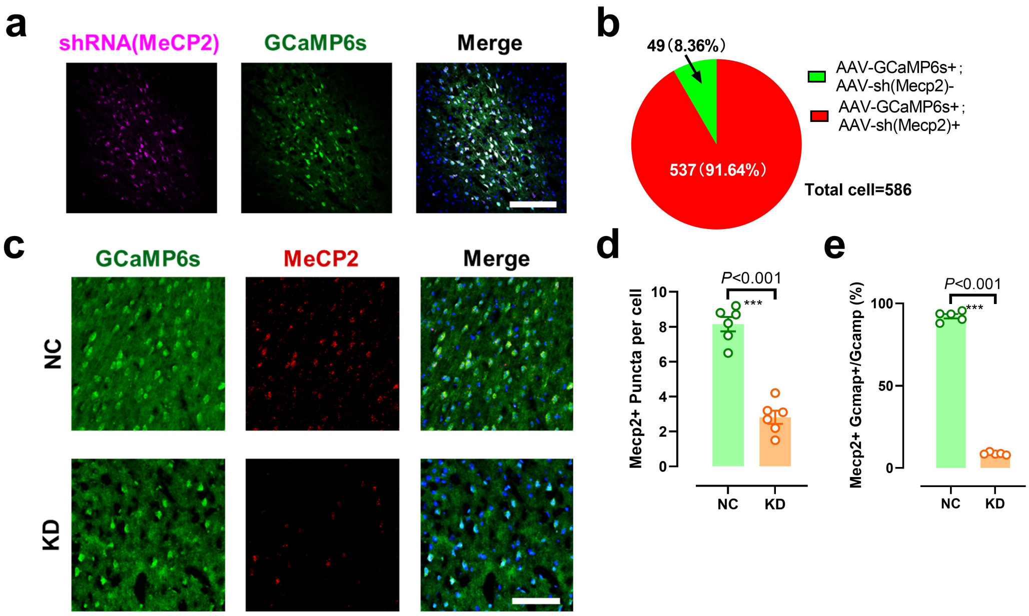

Figure S14. Cell-specificity of MeCP2 gene knockdown. (a) Fluorescent imaging of AAV-shRNA(MeCP2) and AAVGCaMP6s in mouse mPFC. Scale bar, 250 μm. **(b)** Among all cells infected with GCaMP6, more than 91% cells also expressed shRNA(MeCP2). **(c)** RNA Scope for the co-localization of MeCP2 gene transcript within GCaMP6s-positive cells. Scale bar, 150 μm. **(d-e)** The gene knockdown (KD) using shRNA(MeCP2) remarkably decreased the overall MeCP2 gene expression **(d)** and in GCaMP6s+ cells **(e)**. Two-sample unpaired *t*-test, **(d)**, $t(10)=9.608$, $P<0.001$; **(e)**, $t(10)=59.08$, $P<0.001$. $N=6$ mice per group in **(d-e)**. ***, $P<0.001$. All data were presented as mean±sem.
